# Supplementary material for: Efficacy of processed amaranth-containing bread compared to maize bread on hemoglobin, anemia and iron deficiency anemia prevalence among two-to-five year-old anemic children in Southern Ethiopia: A cluster randomized controlled trial
Source: PLoS One. 2020 Sep 28;15(9):e0239192. doi: 10.1371/journal.pone.0239192 (PMC7521750; doi:10.1371/journal.pone.0239192)
Supplement: S1 Table — (DOCX) [file pone.0239192.s001.docx]

**S1 Table.** Main outcome variables result of intra-class correlation coefficient

| **Eight groups** | **Intra class coefficient** | **Confidence interval** | **P-value** |
| --- | --- | --- | --- |
| **Anemia end-line** | 0.04 | 0.002-0.51 | 0.19 |
| **Hemoglobin end-line** | 0.09 | 0.01-0.58 | 0.11 |
| **Hemoglobin baseline** | 0.01 | 8.35E-07 | 0.41 |
| **Ferritin baseline** | 0.05 | 0.003 - 0.51 | 0.16 |
| **Ferritin end-line** | 0.03 | 0.001 - 0.6 | 0.25 |
