# Supplementary material for: Efficacy of processed amaranth-containing bread compared to maize bread on hemoglobin, anemia and iron deficiency anemia prevalence among two-to-five year-old anemic children in Southern Ethiopia: A cluster randomized controlled trial
Source: PLoS One. 2020 Sep 28;15(9):e0239192. doi: 10.1371/journal.pone.0239192 (PMC7521750; doi:10.1371/journal.pone.0239192)
Supplement: S2 Table — (DOCX) [file pone.0239192.s002.docx]

**S2 Table.** Comparing the distribution between complete-case and missed-case.

|  | | **Completed arms 82 (%)** | | **Total** | **Missed arms 18 (%)** | | **Total** |
| --- | --- | --- | --- | --- | --- | --- | --- |
|  |  | **Maize=41** | **Amaranth=41** |  | **Maize=9** | **Amaranth=9** |  |
| **Child sex** | Boy | 19 (46.3) | 25 (61.0) | 44 (53.7) | 3 (30.0) | 4 (50.0) | 7 (38.9) |
|  | Girl | 22 (53.7) | 16 (39.0) | 38 (46.3) | 7 (70.0) | 4 (50.0) | 11 (61.1) |
| **Child age in months (constant)** | | 38 (34-42) | 36 (33-40) | 37 (35-40) | 35 (23-41) | 46 (28-48) | 37(31-43) |
| **Electricity access** | No | 10 (24.4) | 12 (29.3) | 22 (26.8) | 2 (20.0) | 2 (25.0) | 4 (22.2) |
|  | Yes | 31 (75.6) | 29 (70.7) | 60 (73.2) | 8 (80.0) | 6 (75.0) | 14 (77.8) |
| **Owns household land** | No | 25 (61.0) | 29 (70.7) | 54 (65.9) | 7 (70.0) | 6 (75.0) | 13 (72.2) |
|  | Yes | 16 (39.0) | 12 (29.3) | 28 (34.1) | 3 (30.0) | 2 (25.0) | 5 (27.8) |
| **Owns domestic animal** | No | 23 (56.1) | 24 (60.0) | 47 (58.0) | 6 (60.0) | 7 (87.5) | 13 (72.2) |
|  | Yes | 18 (43.9) | 16 (40.0) | 34 (42.0) | 4 (40.0) | 1 (12.5) | 5 (27.2) |
| **Current breast feeding** | Yes | 16 (39.0) | 15 (36.6) | 31 (37.8) | 5 (55.6) | 4 (50.0) | 9 (52.9) |
|  | No | 25 (61.0) | 26 (63.4) | 51 (62.2) | 4 (44.4) | 4 (50.0) | 8 (47.1) |
| **Iron supplement during pregnancy** | No | 26 (63.4) | 23 (56.1) | 49 (59.8) | 8 (80.0) | 7 (87.5) | 15 (83.3) |
|  | Yes | 15 (36.6) | 18 (43.9) | 33 (40.2) | 2 (20.0) | 1 (12.5) | 3 (16.7) |
| **Vaccine completed (mother’s response)** | No | 7 (17.1) | 4 (9.8) | 11 (13.4) | 1 (10.0) | 1 (12.5) | 2 (11.1) |
|  | Yes | 34 (82.9) | 37 (90.2) | 71 (86.6) | 9 (90.0) | 7 (87.5) | 16 (88.9) |
| **Diarrhea in the past 15 days** | No | 27 (65.9) | 25 (61.0) | 52 (63.4) | 5 (50.0) | 5 (62.5) | 10 (55.6) |
|  | Yes | 14 (34.1) | 16 (39.0) | 30 (36.6) | 5 (50.0) | 3 (37.5) | 8 (44.4) |
| **Cough in the past 15 days** | No | 32 (78.0) | 34 (82.9) | 66 (80.5) | 6 (60.0) | 6 (75.0) | 12 (66.7) |
|  | Yes | 9 (22.0) | 7 (17.1) | 16 (19.5) | 4 (40.0) | 2 (25.0) | 6 (33.3) |
| **Hospital admission since birth** | No | 35 (85.4) | 36 (87.8) | 71 (86.6) | 10 (100.0) | 8 (100.0) | 18 (100.0) |
|  | Yes | 6 (14.6) | 5 (12.2) | 11 (13.4) | 0 | 0 |  |
| **Mother aware of iron-rich foods** | No | 36 (87.8) | 40 (97.6) | 76 (92.7) | 8 (80.0) | 7 (87.5) | 15 (83.3) |
|  | Yes | 5 (12.2) | 1 (2.4) | 6 (7.3) | 2 (20.0) | 1 (12.5) | 3 (16.7) |
| **Mother’s occupation** | Unemployed | 28 (68.3) | 26 (63.4) | 54 (65.9) | 5 (50.0) | 4 (57.1) | 9 (52.9) |
|  | Day laborer | 5 (12.2) | 12 (29.3) | 17 (20.7) | 4 (40.0) | 1 (14.3) | 5 (29.4) |
|  | Government /non- government | 8 (19.5) | 3 (7.3) | 11 (13.4) | 1 (10.0) | 2 (28.6) | 3 (17.6) |
| **Father’s occupation** | Unemployed | 6 (14.6) | 8 (19.5) | 14 (17.1) | 3 (30.0) | 0 (0.0) | 3 (17.6) |
|  | Day laborer | 15 (36.6) | 21 (51.2) | 36 (43.9) | 3 (30.0) | 4 (57.1) | 7 (41.2) |
|  | Government/non-government | 20 (48.8) | 12 (29.3) | 32 (39.0) | 4 (40.0) | 3 (42.9) | 7 (41.2) |
| **Household dietary diversity** | Low | 13 (32.5) | 13 (31.7) | 26 (32.1) | 5 (55.6) | 1 (14.3) | 6 (37.5) |
|  | Medium | 19 (47.5) | 17 (41.5) | 36 (44.4) | 2 (22.2) | 3 (42.9) | 5 (31.3) |
|  | High | 8 (20.0) | 11 (26.8) | 19 (23.5) | 2 (22.2) | 3 (42.9) | 5 (31.3) |
| **Child dietary diversity** | Low | 32 (80.0) | 30 (73.2) | 62 (76.5) | 6 (60.0) | 4 (57.1) | 10 (58.8) |
|  | Medium | 6 (15.0) | 9 (22.0) | 15 (18.5) | 3 (30.0) | 3 (42.9) | 6 (35.3) |
|  | High | 2 (5.0) | 2 (4.9) | 4 (4.9) | 1 (10.0) | 0 (0.0) | 1 (5.9) |
| **Household food security** | Food secure | 15 (36.6) | 13 (31.7) | 28 (34.1) | 0 (100.0) | 3 (100.) | 3 (16.7) |
|  | Mild food- security | 4 (9.8) | 6 (14.6) | 10 (12.2) | 2 (20.0) | 2 (25.0) | 4 (22.2) |
|  | Moderate food- insecurity | 7 (17.1) | 8 (19.5) | 15 (18.3) | 2 (20.0) | 1 (12.5) | 3 (16.7) |
|  | Severe food- insecurity | 15 (36.6) | 14 (34.1) | 29 (35.4) | 6 (60.0) | 2 (25.0) | 8 (44.4) |
| **HAZ** | Normal height | 24 (58.5) | 16 (39.0) | 40 (48.8) | 6 (60.0) | 3 (37.5) | 9 (50.0) |
|  | Stunting | 17 (41.5) | 25 (61) | 42 (51.2) | 4 (40.0) | 5 (62.5) | 9 (50.0) |
| **WHZ** | Normal | 37 (97.4) | 37 (94.9) | 74 (96.1) | 9 (100.0) | 6 (75.0) | 15 (88.2) |
|  | Overweight | 1 (2.6) | 1 (2.6) | 2 (2.6) | 0 (0) | 1 (12.5) | 1 (5.9) |
|  | Wasting | 0 (0.0) | 1 (2.6) | 1 (1.3) | 0 (0) | 1 (12.5) | 1 (5.9) |
| **Household income per day** | Less than 1.9$ | 30 (73.2) | 35 (85.4) | 65 (79.3) | 7 (70.0) | 6 (85.7) | 13 (72.2) |
|  | Greater than 1.9$ | 11 (26.8) | 6 (14.6) | 17 (20.7) | 3 (30.0) | 1 (14.3) | 4 (27.8) |
