# Supplementary material for: Efficacy of processed amaranth-containing bread compared to maize bread on hemoglobin, anemia and iron deficiency anemia prevalence among two-to-five year-old anemic children in Southern Ethiopia: A cluster randomized controlled trial
Source: PLoS One. 2020 Sep 28;15(9):e0239192. doi: 10.1371/journal.pone.0239192 (PMC7521750; doi:10.1371/journal.pone.0239192)
Supplement: S1 Appendix — (DOCX) [file pone.0239192.s003.docx]

# Consent form for cross sectional study

Locality Name_____________ Village Name ________________________

Name of Household Head____________________ Name of Mother_________________

Name of Child_ ______________________ Cluster Number __________________

Household Identity Number________________ Name of Supervisor______________________

How are you? My name is _____________________Your house is selected randomly for this interview .The purpose of this interview is to collect base line data for the next experimental study for the evaluation of amaranth grain on the iron status of children in the age of 24-59 months and which factors that are related to that. The eligible volunteer will be engaged in the study if they fulfill the criteria. Decision on your involvement will be made by you and only you. If you are willing to participate, you will be expected to realize the following requirement you are required to give us information about you, your house hold and about child, the child weight and height will be measured and blood will be collected from your child to know the child anemia status. Should you here say that around 1/3 from the survey will be asked to participate in a nutrition study? Or should you wait?

**Risk** the child will fell mild pain during blood collection and there is no further physical or psychological risk expected being involved in the study

**Benefit** You have the right to know the finding of the study, you will be given experts advice about anemia and iron rich foods. The result which produced from your participation will help to fight iron deficiency anemia in the nation.

**Confidentiality** Yours and your Childs information will only be used for the purpose of the study. You and your child will not be personally identified in the study report without your will.

**Participation** You have to know that your participation is largely based on your willingness and approval. You have the right to say “no” and not participate in the study. You will not be penalized if you decide not to participate. If you wish to withdraw from this study you can do that at any time

**Questions:** you can ask any question about this study

**Confirmation of agreement:** I have read the consent form /the interviewer has read the consent form .I have understand the aim of the study and the things that I have to do if I agreed to participate in the study .I know that my participation is based on my will and have right not to do so, if I do not want to participate .please tell us if you agree or not

Yes ______________ No______________

Mothers name _________________signature _____________

Interviewer signature ______________________________

Date _____________________________

Thank you for your willingness to participate in this study

**Xaphoomuna babaxitino xiinxello sumuumme**

**Title:** Preventing iron deficiency anemia: Evaluation of amaranth grain supplementation to 24.0-59.9 month old children in southern Ethiopia, a randomized controlled trial

**Teessote Su’ma ______________________Olluu summa_______________________**

**Mini maate anni su’ma _____________________________Amate Su’ma _______________________**

**Qaaqquu su’ma_________________Qachu Kiiro_________________________**

**Minu Kiiro__________________Sooreessu Suma __________________________**

**Keereholla ? Heeshsho hittoti? Summa’ya __________________________________ yaamameemo.**

Ate mine xinxallote qorsha assate doodhinomo tenne xiinxallo qorshi korkaati. Raffote yinayi shaanina gumisi giddo afentano ayironete yinayiti Qaaqqulleho yaano 24-59 agani daaimira uyitanno horronna mannu tenne horo uyitanno sagale horonsi’rate roso xiinxallateeti .Xiinxallonkera mahoye yaanohuna.Hasiisannore wonshannohu assineemo xiinxallora sumuu yateni kaalannonke.Tenne yoo mahoye yaattohu umikki fajjonni atenna ate callaho. Sumuu yiittoro koonni aantino xa’mora helaale ikkitino dawaro dawaratonkehu minikki maate lainohunnina qaaqqoki lainnohuniti. qaaqqu ayirrena hajjassi seendille keenneemo. anemiyu (mundeete anje) xissonni fayyo ikassina loophosi buuxate shiima mundee haa’nemo. **Hullullo:** Mundee haa’nayi woyite qaaqqoho xissote gederi maccishamawo .ikkollana kayiinni bisisi ananna angoolessi aana kalaqamemo qarri dino. **Horo:** Tenne xiinxaallo hasatto maatiro afate mafte (feceleeqo) noohe. Aneemiyu(mundeete anje) xisso lainohunnina ayirene uyitano sagale lainohunni rosicho uyiinannihe. Ati ledonke loosakinni kainohunni qaaqquullu aana iilitanota ayirenete anjenni dagannota mundeete anje gargarate dandineemo. **Afa nooheti** : Atena qaaqqikki dawaro horo uyitennohu Tenne xiinxallo callateeti. Atenna qaaqqikki fajo nookiha ki’ne ayima ayiino afara didandaanno. **Beeqqa:** Ate beqqa tenna xinxallora uyiitanno horo buuxxe affe ‘’mahoooye’’ woy “gimbi” yaate dandaatota. Maahoye yaa giwittoro gadadishshuni/wolqateni mahoye yiisiisannohehu nooikkihura . Debeeqqeeemo yaa dandaatto .Hanaffeno urrisemmo yiitoro ayii yannaranno uurisa dandaatto. **Xamo:**Xiinxallo lainohunni ayii xa’mono xa’ma dandaatto Sumuu yaate buuxo: Xiinxallote borro mabawoomo xiinxallote borro xa’maanchu seeke mabawinoe. Xiinxallote heddonna assinayire baala buuxxoomo. Beeqqate summuu yoommo. Xiinxallo assineyihu umiya fajjonnina beeqqonni ikino dafira tashi yiiero beeqqanna tashi yaa hoogiero agura dandemota buxoommo.

Kulie henni mahoyenso gimbi yaato?

**Mahooyye ____________________________**

**Gimbi_____________________________________**

**Amate su’ma ________________________________Malaate____________________________**

**Xamaanchu su’ma___________________________________**

**Barra___________________________________**

**Tenne xiinxallo beeqqate unikki fajjonni sumuu yoottohure lowo geeshsha galaxineemo**

**SURVEY QUESTIONNAIRE - PREVENTING IRON DEFICIENCY ANEMIA IN ETHIOPIA
*DATE OF INTERVIEW |____|____| Day |___|___| Month |__|__||__|__| Year***

***TIME STARTED |__ |__| Hour |__|__| Minutes***

***TIME ENDED |__ |__| Hour |__ |__ | Minutes***

**INTERVIEWER NAME ______________________________________**

**SUPERVISOR NAME ________________________________________**

**CHECKED BY ______________________________________________**

**ENTERED BY _______________________________________________**

| **LOCALITY NAME_____________________________________________________________**  **VILLAGE NAME _____________________________________________________________**  **NAME OF HOUSEHOLD HEAD____________________________________________________**  **NAME OF MOTHER __________________________________________________________**  **NAME OF CHILD ____________________________________________________________**  **HOUSEHOLD IDENTITY NUMBER_________________________________________________**  **KEBELE CODE_________________________________________________________** |
| --- |

**Part I**

**Socio demographic characteristics of the mother or care giver of the child**

**Ask the caregiver to find the vaccination card (cross-check all information with the card)**

**እናትየውን የክትባት ካርድ ጠይቀህ/ሽ የምትሰ ጠውን መልስ ከካርዱ ጋር አስተያይ/ዪ**

| **No** | **Question** | | |  | |  | | | |  | |  |
| --- | --- | --- | --- | --- | --- | --- | --- | --- | --- | --- | --- | --- |
| 101 | **How long have you been living here as household/family? Put in year** | | |  | |  | | | |  | |  |
|  | Tenne mini maate ledo megegshi yanna heerita? Diro KUlie | | |  |  |  |  |  |  |  |  |  |
|  | ቤተሰብህ/ሽ ለምን ያህል ጊዜ እዚህ ቆያቸሁ; በአመት አስቀምጥ/ጪ (ከ 6 ወር በታች ከሆነ =0 ፤ ከ 6 ወር በላይ ከሆነ =1 አስቀመጥ/ጭ) | | |  |  |  |  |  |  |  |  |  |
| 102 | **Did your household planed to live here for the next one year?** | | |  | |  | | | |  | |  |
|  | Danno diro konne heerate hedo noohe? | | | 0. [_] No | | 0. [_] Dinoe | | | | 0. [_] የለውም | |  |
|  | ቤተሰብሸ ለሚቀጥለው 1 አመት እዚህ የመቆየት እቅድ አላችሁ? | | | 1. [_] Yes | | 1. [_] Nooe | | | | 1. [_]አለው | |  |
| 103 | **How old is this child? (age in month) and date of birth in GC and EC** | | |  | |  | | | |  | |  |
|  | 3a. Qaaqqu ilamino barra(Itiyopiyu kiironni) | | |  | | ____,_____,___ | | | | ___,_____,___ | |  |
|  | ህጻኑ የተወለደበት ቀን (በኢትዩጰያ አቆጣጠር) | | |  | |  |  |  |  |  |  |  |
|  | 3b. Illamino barra (aroyopu kiirronni) | | |  | | _____,_____,___ | | | | _____,_____,___ | |  |
|  | ህጸኑ የተወለደበት ቀን (በአውሮፓ) አቆጣጠር | | |  | |  |  |  |  |  |  |  |
|  | 3c. Ilamino barra afamannoki haikkiro qaaqqu diro agaanunni | | |  | | _________ | | | | ___________ | |  |
|  | የተወለደበት ቀን የማይታወቅ ከሆነ የልጁ እድሜ በወር | | |  | |  |  |  |  |  |  |  |
| 104 | **How old are you? (mother age in years)** | | |  | |  | | | |  | |  |
|  | Dirikki meeho? Kiirotenni kulie. | | |  | |  | | | |  | |  |
|  | የእናትየው ዕድሜ ስንት ነው ? (በአመት ) | | |  | |  |  |  |  |  |  |  |
|  | **Is the child a boy or a girl?** | | |  | |  | | | |  | |  |
| 105 | Daaimu Koo/tee? Meyatenso labaho | | | 1. [_] Boy | | 1. [_] Labaaho | | | | [_] 1.ወንድ | |  |
|  | ሕፃኑ ወንድ ነው ሴት? | | | 2. [_] Girl | | 2. [_] Meyaate | | | | [_] 2.ሴት | |  |
| 106 | **What is your relationship with the child?** | | |  | |  | | | |  | |  |
|  | Daaimuua ate fixoomi maati? | | | 1. [_] Mother | | 1.[_]Amate | | | | 1[_] እናት | |  |
|  | አንቺ ለህፃኑ ምኑ ነሸ? | | | 2. [_] Father | | 2.[_]Annaho | | | | 2[_] አባት | |  |
|  |  | | | 3.[_] Step mother /father | | 3.[_]Buddeenu amate | | | | 3[_] እንጀራ አናት/ባት | |  |
|  |  | | | 4.[_] Grand mother/Father | | 4.[_]Ahaahete/ hoho | | | | 4[_] አያት | |  |
|  |  | | | 5. [_] Sister/brother | | 5.[_]Rodoote/Rodooho | | | | 5[_]አህት/ወንድም | |  |
|  |  | | | 6. [_] uncle/aunt | | 6.[_] Aboho/halamete | | | | 6[_] አጉት/አክሰት | |  |
|  |  | | | 7. [_] other specify | | 7.[_]Welere | | | | 7[_]ሌላ/ዘርዝር | |  |
| 107 | **What is your religion** | | |  | |  | | | |  | |  |
|  | Anunoki meati? | | | 1.[_]Orthodox | | 1.[_]Orthodoksete | | | | 1[_] አርቶደክሰ | |  |
|  | ሀይማኖትሸ ምንድን ነው? | | | 2. [_] Protestant | | 2.[_] Protestantete | | | | 2[_] ጴንጤ | |  |
|  |  | | | 3. [_] Catholic | | 3.[_] Katoolikete | | | | 3[_] ካቶሊክ | |  |
|  |  | | | 4. [_] Muslim | | 4.[_] Isilamaho | | | | 4[_] ሙስሊም | |  |
|  |  | | | 5. [_] Traditional | | 5.[_] Budu amanooti | | | | 5[_] ባህላዊ | |  |
|  |  | | | 6. [_] Other specify | | 6.[_] Wolere | | | | 6[_] ሌላ/ዘርዝር | |  |
| 108 | **What is your marital status?** |  | |  | |  | |  | |  | |  |
|  | Mine kalaqirooto/ta? | | | 1.[_]Single | | 1.[_]Minaamedti | | | | 1[_] ያገባ | |  |
|  | የጋብቻ ሁኔታሽ/ህ ምን ይመስላል? | | | 2. [_] Married | | 2.[_]Qeedhichchaho | | | | 2[_] ያለገባ | |  |
|  |  | | | 3. [_] Divorced | | 3.[_] Fateherawase | | | | 3[_] ተራርቆ የሚኖር | |  |
|  |  | | | 4. [_] Widowed | | 4.[_] Tiroomo/ma | | | | 4[_] የተፋታ/ች | |  |
|  |  | | | 5. [_] Separated | | 5.[_]Shirroomo/ma | | | | 5[_] የሞተባት/በት | |  |
| 109 | **How many years of completed school does the mother have?** | | |  | |  | |  | |  | |  |
|  | Rosu aana mee diro sayisootta | | |  | |  | | | |  | |  |
|  | በትምህርት ላይ ምን ያህል አመት አሳልፈሻል? | | |  | |  |  |  |  |  |  |  |
| 110 | **How many years of completed school does the father have?** | | |  | |  | |  | |  | |  |
|  | Annu rosu mine mee dirosayisino | | |  | |  | | | |  | |  |
|  | የህፃኑ አባት በትምህርት ቤት ምን ያሀል አመት አሳልፏል? | | |  | |  |  |  |  |  |  |  |
| 111 | **What is the total number of people living in your household?** | | |  | |  | |  | |  | |  |
|  | Tenne mini maate eiddo moo manni kirro meeho? | | |  | |  | | | |  | |  |
|  | በዚህ ቤት ውስጥ ምን ያህል ሰው ነው የሚኖረው? | | |  | |  |  |  |  |  |  |  |
| 111A | **A. How many are younger than 5 year?** | | |  | |  | |  | |  | |  |
|  | Ontu dirii worihu meeho? | | |  | |  | | | |  | |  |
|  | ከ5 አመት በታች ምን ያህል ናቸው? | | |  | |  |  |  |  |  |  |  |
| 111B | **How many are 5 and older but younger than 18year?** | | |  | |  | |  | |  | |  |
|  | Ontu dirii alihuna 18 dirii worihu nee mannati? | | |  | |  | | | |  | |  |
|  | በ5 አመትና በ18 መከከል ምን ያህል ናቸው? | | |  | |  |  |  |  |  |  |  |
| 111C | **How many are 19 up to 59year?** | | |  | |  | |  | |  | |  |
|  | 19-59diri geesha nee mannaati? | | |  | |  | | | |  | |  |
|  | ከ19-59 አመት መካከል ምን ያህል ናቸው? | | |  | |  |  |  |  |  |  |  |
| 111D | **How many are older than 60 year?** | | |  | |  | |  | |  | |  |
|  | 60 diri alihu meeho? | | |  | |  | | | |  | |  |
|  | ከ60 አመት በላይ ምን ያህል ናቸው? | | |  | |  |  |  |  |  |  |  |
|  | | | | | | | | | | | |  |
| **Part 2 Question on Economic status of the house hold** | | | | | | | | | | | | |
| Kirro 2 Mini manni maatete jirote bikka | | | | | | | | | | | | |
| ክፍል 2. የቤተሰብ ምጣኔ ሀብት መለኪያ መጠይቅ | | | | | | | | | | | | |
| 201 | **What is the mother occupational status?** | |  | |  | | | |  | | | |
|  | Amote loosi hiitooho? | | 1.[_] Unemployed | | 1.[_]Loosu dinooho | | | | 1[_]ሰራ የሌለው | | | |
|  | የእናትየው ዋና ስራ ምንድን ነው? | | 2. [_] Day laborer | | 2.[_]Barru loosasicho | | | | 2[_]የቀን ሰራተኛ | | | |
|  |  | | 3. [_] Farmer | | 3.[_] Baatote looso | | | | 3[_]ገበሬ | | | |
|  |  | | 4. [_] Merchant | | 4.[_] Dadalanchoho | | | | 4[_]ነጋዴ | | | |
|  |  | | 5. [_] NGO employed | | 5.[_]Mengistati looso | | | | 5[_]የድርጅት | | | |
|  |  | | 6.[_]Government employed | | 6.[_]Manaisete looso | | | | 6[_]የመንግሰት | | | |
|  |  | | 7. [_] Student | | 7.[_]Rosaanchoho | | | | 7[_]ተማሪ | | | |
|  |  | | 8.[_]Other specify | | 8.[_]wolere xawisi | | | | 8[_]ሌላ ካለ ግለፅ | | | |
| 202 | **What is the Father occupational status?** | |  | |  | | | |  | | | |
|  | Annu Loosi hiittooho? | | 1.[_] Unemployed | | 1.[_]Loosu dinooho | | | | 1[_]ሰራ የሌለው | | | |
|  | የአባትየው የስራ ሁኔታ? | | 2. [_] Day laborer | | 2.[_]Barru loosasicho | | | | 2[_]የቀን ሰራተኛ | | | |
|  |  | | 3. [_] Farmer | | 3.[_] Baatote looso | | | | 3[_]ገበሬ | | | |
|  |  | | 4. [_] Merchant | | 4.[_] Dadalanchoho | | | | 4[_]ነጋዴ | | | |
|  |  | | 5. [_] NGO employed | | 5.[_]Mengistati looso | | | | 5[_]የድርጅት | | | |
|  |  | | 6. [_] Government employed | | 6.[_]Manaisete looso | | | | 6[_]የመንግሰት | | | |
|  |  | | 7. [_] Student | | 7.[_]Rosaanchoho | | | | 7[_]ተማሪ | | | |
|  |  | | 8.[_]Other specify | | 8.[_]wolere xawisi | | | | 8[_]ሌላ ካለ ግለፅ | | | |
| 203 | **What was last month income?** | |  | |  | | | |  | | | |
|  | Sai aganira megeeshi afidhinoonni | |  | |  | | | |  | | | |
|  | ባለፈው ወር ቤተሰቡ ያሰገባው ወራዊ ገቢ ምን ያህል ነበር? | |  | |  |  |  |  |  |  |  |  |
|  | **What is the average yearly income of the household?** | |  | |  | | | |  | | | |
| 204 | Dirunnit mereerima eone megeeshshaatic? | |  | |  | | | |  | | | |
|  | በአማካይ የቤተሰቡ አመታዊ ገቢ ምን ያህል ነበር? | |  | |  |  |  |  |  |  |  |  |
| 205 | **How much do you save yearly?** | |  | |  | | | |  | | | |
|  | Dirruni megeeshshi woxe suuqisidhinenni? | |  | |  | | | |  | | | |
|  | በአመት ምን ያህል ትቆጥባላችሁ? | |  | |  |  |  |  |  |  |  |  |
| 206 | **House hold facility : Do you have any of the following** | |  | |  | | | |  | | | |
|  | Mini meate injo konniaane nori gido kinera noori noo’ne? | | 1.      [_] Electricity | | 1[_] Maabraate | | | | 1[_] ማብራት | | | |
|  | በቤት ውሰጥ ከሚገኙ መገልገያ ቁሶች የትኞቹ አሉሽ?(የሌላትን **0** ሙላ**)** | | 2.      [_] Radio | | 2[_] Radoone | | | | 2[_] ራዲዎ | | | |
|  |  | | 3.      [_]Mobile telephone | | 3[_]Silke | | | | 3[_] ሰልክ | | | |
|  |  | | 4.      [_]Non mobile telephone | | 4[_] Teevizhiine | | | | 4[_] ቴሌሺዥን | | | |
|  |  | | 5.      [_] Television | | 5[_]Qiissaasinchu | | | | 5[_] ፍሪጅ | | | |
|  |  | | 6.      [_] computer | | 6[_]Maabiraatete | | | | 6[_] የማብራት ምድጃ | | | |
|  |  | | 7.      [_] refrigerator | | 7[_] Compitere | | | | 7[_] ኮምፒውተር | | | |
|  |  | | 8.      [_] electric stove | | 8[_] Sayiikile | | | | 8[_] ሳይክል | | | |
|  |  | | 9.      [_] Motor bike | | 9[_]Motorete sayikle | | | | 9[_] ሞተር ሳይክል | | | |
|  |  | | 10[_] Car | | 10[_] መኪና | | | | 10[_] መኪና | | | |
|  |  | | 11[_] Other specify | | 11[_] ሌላ ካለ ግለፅ | | | | 11[_] ሌላ ካለ ግለፅ | | | |
| 207 | **Does the household own any agricultural land?** | |  | |  | | | |  | | | |
|  | Konni mini maatera umi’ne baato loosidhinenniti noo’ne? | | 0. [_] No | | 0. [_] Dinoe | | | | 0[_] የለውም | | | |
|  | የዚህ ቤት ባለቤት የእርሻ መሬት አለው? | | 1. [_] Yes | | 1. [_] Nooe | | | | 1[_] አዎ | | | |
| 208 | **How many (LOCAL UNITS) of agricultural land do this household own?** | |  | |  | | | |  | | | |
|  | Allanne halaligne mepeeshite (Kine keeninni) Akine umine baattoti? | |  | |  | | | |  | | | |
|  | የእርሻ መሬት ካለው ምን ያህል መሬት ነው ያለው (በባህለዊ መለኪያ አስቀምጥ ) | |  | |  |  |  |  |  |  |  |  |
| 209 | **Does the house hold produce any yield with this land** | |  | |  | | | |  | | | |
|  | Baatone loosine guma afidhinenni? | | 0. [_] No | | 0. [_]Diafineeno | | | | 0[_] የለውም | | | |
|  | በዚህ መሬት የምታመርቱት ምርት አለ? | | 1. [_] Yes | | 1. [_]Afineeno | | | | 1[_] አዎ | | | |
| 210 | **If yes what do you produce and how much (put zero if not)** | |  | |  | | | |  | | | |
|  | Afidhinennihe ikkino baattoneana loosiinennihu maati? Mageeshshua afidhinanni? | |  | |  | | | |  | | | |
|  | **ከሚከተለው ምርት ውስጥ ምን ያህል ታመርታላችሁ (ካላመረቱ 0 አስቀምጥ)** | |  | |  |  |  |  |  |  |  |  |
|  | Inset? (root) | |  | | Weese | | | | እንሰት | | | |
|  | Maize? (quintal) | |  | | Badala | | | | በቆሎ | | | |
|  | Cabbage?(lood) | |  | | Shaana | | | | ጎመን | | | |
|  | Potato? (quintal) | |  | | Dinnichha | | | | ድንች | | | |
|  | Chilli? (quintal) | |  | | Shama barbare | | | | ቃሪያ | | | |
|  | Sugercan?(load) | |  | | Shoonkoora | | | | ሸነኮራ ዐገዳ | | | |
|  | Tomato? (box) | |  | | Timatime | | | | ቲማቲም | | | |
|  | Banana? (load) | |  | | Muuze | | | | ሙዝ | | | |
|  | Avocado? (quintal) | |  | | Awukado | | | | አቮካዶ | | | |
|  | Mango? (quintal) | |  | | Maango | | | | ማንጎ | | | |
|  | Kchat? (load) | |  | | Chaate | | | | ጫት | | | |
|  | Other specify | |  | | Welere | | | | ሌላ ካለ ግለፅ | | | |
|  |  |  |  | |  |  |  |  |  |  |  |  |
| 211 | **Does your house hold own any domestic animal** | |  | |  | |  | |  | |  | |
|  | Konni mini maatera mini saado no? | | 1. [_] Yes | | 0. [_] Dinoe | | | | 0 [_].አይደለም | | | |
|  | የቤት እንስሳት አላችሁ? | | 2. [_] No | | 1. [_] Nooe | | | | 1 [_].አዎ | | | |
| 212 | **How much of the following do you have?**  Afidhinoonniha ikkiro hiite saada? Megessha? | | Chiken | | 1.Lukkicho____ | | | | 1. ዶሮ ____ | | | |
|  | የቤት እንሰሳ ካለችሁ ከሚከተሉት ውስጥ ምን ያህል ዐላችሁ?**(የሌለውን 0 አስቀምጥ)** | | Goat | | 2. Meicho_____ | | | | 2. ፍየል____ | | | |
| ዝለል  Skip |  | | Sheep | | 3.Gereewo____ | | | | 3. በግ ____ | | | |
|  |  | | Ox | | 4.Bootta_______ | | | | 4. በሬ ________ | | | |
|  |  | | Cow | | 5.Lalo______ | | | | 5. ላም ____ | | | |
|  |  | | Donkey | | 6.Harricho____ | | | | 6. አህያ ____ | | | |
|  |  | | Other specify | | 7.wolere xawisi_______ | | | | 7. ሌላ ዘርዝሪ/ር____ | | | |
|  |  | |  | |  | | | |  | | | |
| 213  ዝለል  Skip | **If yes for question number 211 Do you feed animal products for your children** | |  | |  | | | |  | | | |
|  | Saedate winni afidhi nannire daaimaho uyitinnan? | | 0. [_] No | | 0. [_] Dinoe | | | | 0. [_] አላበላም | | | |
|  | የራሰሸን የከብቶች ተዋፅኦ ለልጆችሸ ታበያለሸ? | | 1. [_] No | | 1. [_] Nooe | | | | 1. [_] አዎ | | | |
| 214 | **What type of latrine do you have** | |  | |  | | | |  | | | |
|  | Shumate mini hittoohu noone? | | 1. [_] no facility/bush/field | | 1[_]Dinoe | | | | 1[_]የለንም | | | |
|  | ምን አይነት ሽንት ቤት ነው ያላችሁ? | | 2. **[_]** composting toilet | | 2[_]Irshu giddo | | | | 2[_]የማሳ ውሰጥ | | | |
|  |  | | 3. [_] open pit | | 3[_]Haqqunnabushshunni calla tu’nooni | | | | 3[_]ክፍት ጉድጎድ | | | |
|  |  | | 4. [_] pit latrine with slab | | 4[_]Simmintoteni loonsoonnishuma mine. | | | | 4[_]የወለል ልባስ ያለው የጉድጎድ ሸንት ቤት | | | |
|  |  | | 5. [_] ventilated improved pit latrine (vip) | | 5[_]Tuubbotenni foolanno shuamte mine. | | | | 5[_]የተሻሻለ የጉድጎድ ሽንት ቤት | | | |
|  |  | | 6.[_] flush or pour flush toilet | | 6[_]Wiyiinni loosanno shumate mine. | | | | 6[_]በውሀ የሚሄድ ሸንት ቤት | | | |
| 215 | **What is the main source of drinking water for your house hold** | |  | |  | |  | |  | |  | |
|  | Waa horonsidhinonnihu maminniti? | | 1.   [_]unprotected well/spring | | 1[_]Huxxinoonnkki buichcho | | | | 1[_]ካልተከለለ የከርሰ ምድርውሀ | | | |
|  | የመጠጥ ውሀ በዋናነት ከየት ነው የምትጠቀሙት? | | 2. [_]protected spring/well | | 2[_]Huxxinoonni buichcho | | | | 2[_]ከተከለለ የከርሰ ምድር ውሀ | | | |
|  |  | | 3. [_]tanker truck | | 3[_]Rottote giddo kuusanino waa | | | | 3[_]ከማጠራቀሚያ | | | |
|  |  | | 4. [_]public tap/standpipe | | 4[_]Olluu horons’rano 5[_]waa Gibbete giddo | | | | 4[_]ከህዝብ ቧንቧ | | | |
|  |  | | 5. [_]piped into dwelling | | 5[_]Waa Gibbete giddo | | | | 5[_]ግቢ ከገባ ቧንቧ | | | |

| **Part 3 child feeding practice**  **Kiiro 3 Qaaqqu sagalate gara** | |  |  |  |
| --- | --- | --- | --- | --- |
| ክፍል **3** የህፃኑ አመጋገብ ሁኔታ | |  |  |  |
| **No** | **Question** | **Answer** |  | **መልስ** |
| 301 | **Do you feed breast milk to your child?** |  |  |  |
|  | Daaima unuuna qansata? | 1. [_] Yes | 1. [_] Qanseema | 1[_] አዎ |
|  | ህፃኑን ዐሁን ጡት ታጠቢዋለሽ? | 2. [_] No | 2. [_]Diqaanseema | 2[_] አላጠባውም |
| 302 | **If the mom is not breastfeeding now: Did you ever breastfeed your child?** |  |  |  |
|  | Ama unuunna qansitahakkiha ikkiro Qaaqqokk unuuna qansootta | 1. [_] Yes | 1. [_] Qanseema | 1[_] አዎ |
|  | እናትየው ዐሁን ጡት ማታጠባ ከሆነ ልጅሽን ጡት አጥብተሽው ነበር(በፊት) | 2. [_] No | 2. [_]Diqaanseema | 2[_] አላጠባውም |
| 303 | **For how long did you breastfeed your child exclusively before giving any other feeds including water** |  |  |  |
|  | ዐጥብታው ከሆነ ለምን ያህል ጊዜ ነው ጡት ያጠባሽዉ (በወር ዐስቀምጭ/ጥ) |  |  |  |
|  | **When did you start feeding your child with other foods apart from breast milk** |  |  |  |
| 304 | Wole segela karsitakinni unuuna calla mageeshi yanna qansita? |  |  |  |
|  | ለልጅሽ ምንም ምግብ ሳትሰጪ ውሀን ጨምሮ ለምን ያህል ጊዜ ነው ጡት ብቻ የሰጠሽው |  |  |  |
| 305 | **When did you start feeding your child with other foods apart from breast milk** |  |  |  |
|  | Daaimaho sagale aameu dirin hanafita? |  |  |  |
|  | ለህፃኑ ተጨማሪ ምግብ በሰንት ወሩ ጀመርሽለት? |  |  |  |
| 306 | **With what food do you start feeding your child?** | 1[_] Cerial porrage | 1[_]Gidu shirku | 1[_]ከእህል ገንፎ |
|  | Daaimikkira itisa hanafootta sagale maati? | 2[_] Cow milk | 2[_]Saddate adonni | 2[_]በከብት ወተት |
|  | ለህፃኑ ምግብ የጀመርሸለት በምንድን ነው? | 3[_] Fruit juice | 3[_]Muroteni huanlinoni | 3[_]በፍራፍሬ ጭማቂ |
|  |  | 4[_] Other specify | 4[_]Wole kuli | 4[_]ሌላ ጥቀሰ |
| 307 | **What is the number of meals normally taken by the child per day?** |  |  |  |
|  | Daaaimikki barrunni meu dani sagale itanno?\ |  |  |  |
|  | ህፃኑ አሁን በቀን ምንያህል ጊዜ ነው የሚመገበው |  |  |  |
| 308 | **Does child can feed himself/herself** |  |  |  |
|  | Daaimu sagale umisinni saga’la dandaanno? | 1[_]Care giver | 1[_]Manncholaatisannohu | 1[_]ተንከባካቢ ያበላዋል |
|  | ህፃኑ በራሱ መመገብ ይችላል? | 2[_]With assistance | 2[_]Irkotenniteitanohu | 2[_]በዕገዛ ይበላል |
|  |  | 3[_]Self feeding | 3[_]Umosit itannohu | 3[_]ህፃኑ በራሱ ይበላል |
|  | **Who feeds the child?** |  |  |  |
| 309 | Daaimaho sagale ayi itisanno? | 1. [_] Mother | 1. [_] Ama | 1[_] እናት |
|  | ህፃኑን በቋሚነት ማን ነው የሚመግበው? | 2. [_] Father | 2. [_] Annu | 2[_] አባት |
|  |  | 3. [_] Ant /Ancle | 3. [_] Abbu/La’lama | 3[_] እንጀራ አናት/አባት |
|  |  | 4. [_] grandfather /mother | 4. [_] Ahahaahu/he | 4[_] አያት |
|  |  | 5. [_] other specify | 5. [_] Woleho xamisi | 5[_] አህት/ወንድም |
|  |  | 6. [_] Do not know | 6. [_] Diafoommo | 6[_] አጉት/አክሰት |
| 310 | **Does the child had food allergy** |  |  |  |
|  | Daaimaho lagaabbino sagale no? | 1. [_] Yes | 0. [_] Dinoe | 0[_] አዎ |
|  | ህፃኑ የማይስማማው የምግብ አይነት አለ? | 2. [_] No | 1. [_] Nooe | 1[_] የለም |
| 311  ዝለል  Skip | **What is the food allergic for?** |  |  |  |
|  | Qaaqqoho (daaimaho) allifatowokki sagalete dani maati |  |  |  |
|  | ህፃኑ የማይሰማማው የምግበ አይነት ምንድን ነው? |  |  |  |
| 312  ዝለል  Skip | **What was the manifestation of allergic condition?** |  |  |  |
|  | Allifatawokki sagala leelli shashanno malaati maati | 1. [_] Rashes | 1.[_]Hafuro | 1[_] ሸፍታ |
|  | የማይስማማዉን ምግብ ሲመገብ የሚያሳየው ምልክት ምንድን ነው? | 2. [_] Vomiting | 2.[_]Tushiishanno | 2[_] ትውከት |
|  |  | 3. [_] Diarrhea | 3.[_]Deeiishshanno | 3[_] ተቅማጥ |
|  |  | 4. [_] Pain | 4. [_]Xissannosi/se | 4[_] ቁርጠት |
|  |  | 5. [_] Swelling | 5. [_]Fuugisanno | 5[_] እብጠት |
|  |  | 6. [_] Do not know | 6. [_]Welere | 6[_] ሌላጥቀሰ |
| 313  ዝለል  Skip | **What is the nutrition action taken for the allergic condition?** |  |  |  |
|  | Daaimu Lagaabbino sagale itiro woyyeessate uyitinanni sagale maati? |  |  |  |
|  | ለማይስማማው ምግብ ምልክት የተወሰደ እርምጃ ካለ ግለጪ |  |  |  |

| **art 4: 24 Hour Dietary Diversity Questioner** | |  |  |  |  | |
| --- | --- | --- | --- | --- | --- | --- |
| Gaamo 4: 24 Sagalete danixamo | |  |  |  |  | |
| ክፍል:4:በ24 ሰአት ዉስጥ የወሰደውን የምግብ አይነት የሚገልጥ ጥያቄ | |  |  |  |  | |
| **No** | **Type of food** |  |  |  | |  |
| 401 | **I am going to ask you questions about what you fed your baby from the time you woke up yesterday morning till you woke up this morning either separately or combined with other foods.** |  |  |  | |  |
|  | Bero soodo qoxootto wiinni kaitto yannanni hanaffe techo soodo geeshsha aante noo segalla giddo daaimaho itisootto sagale no? Itisoottaha ikkiro me’e higge itisoottoro kulattoe? Itisootto segale no  ዐሁን ህፃኑ ከትላነት ጠዋት ዕስከ ዛሬ ጠዋት የተመገበውን የምግብ ዐይነት ዕጠይቅሻለሁ (ህፃኑ የተመገበውን ቁርስ ፣ምሳ ዕራት ዕንዲሁም በየመሀል የተመገበውን በመጠየቅ የሚስማማውን ቦታ ጥቀስ) |  |  |  | |  |
| 402 | **Did your child eat any porridge or gruel (from what it made)** |  |  |  | |  |
|  | Daaimu sherko woy axmiite saga’lino (mayinni qixxeessinoonniha)? | 0[_] No | 0.[__]Disaga’lino | 0[_] አልተመ ገበም | |  |
|  | ልጅሸ ከማንኛውም የእህል ዘር የተሰራ ገንፎ ወይም ሙቅ ተመግቧል? | 1[_] Yes | 1.[__]Saga’lino | 1[_] ተመግ ቧል | |  |
| 403 | **Bread, pasta, rice, noodles, biscuits, cookies or any other food made from ,oats, maize, barley, wheat, sorghum millet, or other grain? Specify** |  |  |  | |  |
|  | Daabbo,paarta,ruuze,koshoro raino sagale woy ajjunni ,badalatenni ,hayixunni,qamadetenni,bashanqunniy loonsoonni sagale woy wolu quminni,xawisi | 0[_] No | 0.[__]Disaga’lino | 0[_] አልተመ ገበም | |  |
|  | ዳቦ፤ ፓስታ፤ ሩዝ፤ ብሰኩት፤ ኩኪሰ፤ ወይም ማንኛውም ነገር ከአጃ ከበቆሎ ገብሰ፤ ሰንዴ፤ ማሸለ፤ወይም ሌላ አህል ዘር የተሰራ | 1[_] Yes | 1.[__]Saga’lino | 1[_] ተመግ ቧል | |  |
| 404 | **Any food made from teff , like injera ,kita or porridge ?** |  |  |  | |  |
|  | Gaashetenni qqxxeessinoonnic segale buddeena, tima,woy sherko lawinore saga’lino? | 0[_] No | 0.[__]Disaga’lino | 0[_] አልተመ ገበም | |  |
|  | ማንኛውም ምግብ ከጤፍ የተሰራ (እንጀራ፤ ቂጣ፤ ገንፎ) | 1[_] Yes | 1.[__]Saga’lino | 1[_] ተመግ ቧል | |  |
| 405 | **Any white potatoes, white yam?** |  |  |  | |  |
|  | Maxaaxeesla, diinicha, boyina,lawinore saga’lino? | 0[_] No | 0.[__]Disaga’lino | 0[_] አልተመ ገበም | |  |
|  | ማንኛውም ነጭ ድንች፤ ቦይና፤ እንሰት (ማንኛውም ነጭ ስራስር) | 1[_] Yes | 1.[__]Saga’lino | 1[_] ተመግ ቧል | |  |
| 406 | **Any foods made from beans, peas, lentils or pulses** |  |  |  | |  |
|  | Aye segale baqeluuni, atarunni ,qibaatete qumma shumburunni qixxeessinoonni sagale saga’lino? | 0[_] No | 0.[__]Disaga’lino | 0[_] አልተመ ገበም | |  |
|  | ባቂላ፤አተር፤ ምሰር ወይም ሌላ ጥራጥሬ | 1[_] Yes | 1.[__]Saga’lino | 1[_] ተመግ ቧል | |  |
| 407 | **Any nuts or seed such as peanut, sesame or sun flower seeds?** |  |  |  | |  |
|  | Ayee qumma ocholoone coommadda gumma saga’lino | 0[_] No | 0.[__]Disaga’lino | 0[_] አልተመ ገበም | |  |
|  | ኦቾሎኒ (ከአቾሎኒ የተሰራ ማንኛውም ምግብ) | 1[_] Yes | 1.[__]Saga’lino | 1[_] ተመግ ቧል | |  |
| 408 | **Any butter,oil** |  |  |  | |  |
|  | Zayitetenni woy buurumi loonsoonni sagala | 0[_] No | 0.[__]Disaga’lino | 0[_] አልተመ ገበም | |  |
|  | በዘይት ወይም በቅቤ የተሰራ ምግብ | 1[_] Yes | 1.[__]Saga’lino | 1[_] ተመግ ቧል | |  |
| 409 | **Any dark green, leafy vegetables like kale, spinach or amaranth leaves?** |  |  |  | |  |
|  | Haanjarino, daraame ataakiltete daronna xu’naayye lawinore saga’lino? | 0[_] No | 0.[__]Disaga’lino | 0[_] አልተመ ገበም | |  |
|  | ማንኛውም ጥቁር አረንጎዴ አታክልት፤ ቅጠላማ አታክልት እንደ ጎመን፤ ራፎ ወይም ሌላ (ዘርዝር) | 1[_] Yes | 1.[__]Saga’lino | 1[_] ተመግ ቧል | |  |
|  | **Any pumpkin ,carrot, squash or sweet potatoes that are yellow or orange inside** |  |  |  | |  |
| 410 | Giddo bica woy haanjirino baaqulaa, kaaroote woy maxaaxeesha saga’lino? | 0[_] No | 0.[__]Disaga’lino | 0[_] አልተመ ገበም | |  |
|  | ማንኛውም ዱባ፤ ካሮት ቢጫ ሰኳር ድንች ሌላ (ዘርዝር) | 1[_] Yes | 1.[__]Saga’lino | 1[_] ተመግ ቧል | |  |
| 411 | **Any ripe mangoes, papayas?** |  |  |  | |  |
|  | Le’ado mango woy paapaayya saga’lino? | 0[_] No | 0.[__]Disaga’lino | 0[_] አልተመ ገበም | |  |
|  | የበሰለ ማንጎና ፓፓያ | 1[_] Yes | 1.[__]Saga’lino | 1[_] ተመግ ቧል | |  |
| 412 | **Any other fruit or vegetables** |  |  |  | |  |
|  | Wole aye gumma woy akaakilte saga’lino? | 0[_] No | 0.[__]Disaga’lino | 0[_] አልተመ ገበም | |  |
|  | ሌላ ማንኛውም አታክልትና ፍራፍሬ | 1[_] Yes | 1.[__]Saga’lino | 1[_] ተመግ ቧል | |  |
| 413 | **Commercially fortified foods.** |  |  |  | |  |
|  | Ashshagantinota daaimu segale? | 0[_] No | 0.[__]Disaga’lino | 0[_] አልተመ ገበም | |  |
|  | በምግብ በልፅገው የሚሸጡ ምግቦች | 1[_] Yes | 1.[__]Saga’lino | 1[_] ተመግ ቧል | |  |
| 414 | **Any cheese or yogurt?** |  |  |  | |  |
|  | Ayibe/geinto saga’lino? | 0[_] No | 0.[__]Disaga’lino | 0[_] አልተመ ገበም | |  |
|  | አይብና እርጎ | 1[_] Yes | 1.[__]Saga’lino | 1[_] ተመግ ቧል | |  |
| 415 | **Frresh milk** |  |  |  | |  |
|  | Iibbado ado | 0[_] No | 0.[__]Disaga’lino | 0[_] አልተመ ገበም | |  |
|  | ትኩስ ወተት | 1[_] Yes | 1.[__]Saga’lino | 1 [_] ተመግ ቧል | |  |
| 416 | **Any eggs?** |  |  |  | |  |
|  | Ayee quuphe | 0[_] No | 0.[__]Disaga’lino | 0[_] አልተመ ገበም | |  |
|  | እንቁላል | 1[_] Yes | 1.[__]Saga’lino | 1[_] ተመግ ቧል | |  |
| 417 | **Any liver ,kidney, heart or other organ meats** |  |  |  | |  |
|  | Afale,mule,wodana,woy wole godowu giddo maalla saga’lino? | 0[_] No | 0.[__]Disaga’lino | 0[_] አልተመ ገበም | |  |
|  | ጉበት፤ ኩለሊት፤ ልብ ወይም የእንሰሳ የውሰጥ ሰውነት ክፍል | 1[_] Yes | 1.[__]Saga’lino | 1 [_] ተመግ ቧል | |  |
| 418 | **Any beef, pork, lamb, goat, rabbit (wild game meat such as antelope or deer)?** |  |  |  | |  |
|  | Bootu maala, mancheemete maala, gereewo,mellenna hilleessa (wole dubbu saada,goljanna,guru’me lawinore segalino? | 0[_] No | 0.[__]Disaga’lino | 0[_] አልተመ ገበም | |  |
|  | የበሬ ስጋ፤ የበግ/የፍየል ስጋ የአሳማ ስጋ የጥንቸል ወይም ሌላ እንሰሳ | 1[_] Yes | 1.[__]Saga’lino | 1[_] ተመግ ቧል | |  |
| 419 | **Any chicken ,duck or other birds** |  |  |  | |  |
|  | Lukko, daakiyye, woy wole cea maala saga’lino | 0[_] No | 0.[__]Disaga’lino | 0[_] አልተመ ገበም | |  |
|  | ዶሮ፤ እርግብ ወይም የወፍ ስጋ | 1[_] Yes | 1.[__]Saga’lino | 1[_] ተመግ ቧል | |  |
| 420 | **Any fish** |  |  |  | |  |
|  | Ayee qilxi’me | 0[_] No | 0.[__]Disaga’lino | 0[_] አልተመ ገበም | |  |
|  | ማንኛውም አሳና የአሳ ምርት | 1[_] Yes | 1.[__]Saga’lino | 1[_] ተመግ ቧል | |  |
| 421 | **Any soft drink specify** |  |  |  | |  |
|  | Ayee shota agatto agino? Aginoha ikkiro xawisi | 0[_] No | 0.[__]Disaga’lino | 0[_] አልተመ ገበም | |  |
|  | ማንኛውም ለስላሳ መጠጦች | 1[_] Yes | 1.[__]Saga’lino | 1[_] ተመግ ቧል | |  |
| 422 | **Coffee and Tea** |  |  |  | |  |
|  | Buna woy shae agino | 0[_] No | 0.[__]Disaga’lino | 0[_] አልተመ ገበም | |  |
|  | ቡናና ሻይ | 1[_] Yes | 1.[__]Saga’lino | 1[_] ተመግ ቧል | |  |
| 423 | Alcoholic drink |  |  |  | |  |
|  | Diribisanno ago (birra,xesiixella,areqe) | 0[_] No | 0.[__]Disaga’lino | 0[_] አልተመ ገበም | |  |
|  | አልኮል መጠጦች (ቢራ፤ አረቄ፤ ጠጅ፤ጠላ) | 1[_] Yes | 1.[__]Saga’lino | 1[_] ተመግ ቧል | |  |
| 424 | **Other specify** |  |  |  | |  |
|  | Welere |  |  |  | |  |
|  | ሌላ ካለ ጥቀስ |  |  |  |  |  |

**Part 5: 7-Days Food frequency questionnaire for iron rich foods**

Sufetto 5: 7 barri giddo ayirenetenni lattino sagale marri marro

ክፍል 5፤በ7ት ቀን ውስጥ ህጻኑ የወሰደው የምግብ አይነት

|  |  | **Answer the number of the day you eat** |
| --- | --- | --- |
| **No** | **Question** | **መልሱን በቀናት ቁጥር ዐስቀምጥ** |
| 501 | **Now I am going to ask you if you gave the following items at all the last week ending yesterday morning. Please answer yes if you gave it and no if you did not give it And if you did, will you please tell how many times you gave it** |  |
|  | Xa xa’meemohehu sa’u lamala jeefonni kayise be’ro soodo geeshsha saga’lino sagaleeti.itinno sagale “saga’lino” itinnoki sagale ’’di’saga’lino’’ yite me’e marro itinoro xawasi. |  |
|  | እሁን የምጠይቅሸ ልጅሸ ባለፉት 7ቀን ውሰጥ አሰከ ዛሬ ጠዋት የበላውን የምግብ አይነት ነው ለአያንደንዱ መልሰ ቁጥሩን ግለጪ ካልተመገበ ዜሮ መፃፍን አትርሳ(ሺ) |  |
| 502 | **Food made from false banana (Kocho,kita,bula,omolicho,genfo)** |  |
|  | Inset? |  |
|  | እንሰት(ቆጮ፣ ቡላ...)? |  |
| 503 | **Cereal group (maize, barley, wheat, oats, ….)** |  |
|  | Weese (badala,hayiixe |  |
|  | የእህልዘር( በቆሎ፤ ገብስ፤ ስንዴ፤ አጃ፤ ዳጉሳ፤....... |  |
| 504 | **Pulse group (bean,pea,chickpea,….)** |  |
|  | Qixxeessinoonni (baqeluuni, atarunni ,qibaatete qumma shumburunni?) |  |
|  | የጥራጥሬ ዘር( አተር፤ ሸንብራ፤ ባቂላ፤ምስር……) |  |
| 505 | **Teff (ingera,bred,porrage)** |  |
|  | Gaashe |  |
|  | ጤፍ |  |
| 506 | **Peanut** |  |
|  | Ocholoone |  |
|  | ኦቾሎኒ |  |
| 507 | **Dark green vegitables (Kale,green paper,qosta)** |  |
|  | Haanja daro ataakilte shaana, qaariya,Xu’naaye, raafote daro |  |
|  | ጥቁር አረንጎዴ አታክልቶች(ጎመን፤ ቃረያ፤ ጥቁር ጎመን፤ ራፎ ……..) |  |
| 508 | **Tomato, Carrot, watermelon, pumpkin** |  |
|  | Timaatime,karoote, Baaqula |  |
|  | ቲማቲም፣ ካሮት፣ ዱባ |  |
| 509 | **ripe mango, papaya** |  |
|  | Lino mango,Lino pappaayya |  |
|  | የበሰለ ማንጎ፤ የበሰለ ፓፓያ |  |
| 510 | **orange, lemmon,** |  |
|  | Burtukanenna loome |  |
|  | ብርቱካንና ሎሚ |  |
| 511 | **Amarnth leaf or grain** |  |
|  | Raffote daronna guma |  |
|  | የራፎ ቅጠል ወይም ፍሬ |  |
| 512 | **Milk and milk product** |  |
|  | Ado geinto burbuxxo |  |
|  | ወተት፤ እርጎ፤ አይብ |  |
| 513 | **Any food contains butter or oil** |  |
|  | Zayitetenni woy buurumi loonsoonni sagala |  |
|  | በዘይት ወይም በቅቤ የተሰራ ምግብ |  |
| 514 | **Egg** |  |
|  | Quuphphe |  |
|  | እንቁላል |  |
| 515 | **Red meet (sheep, goat, ox**) |  |
|  | Dummo maala (bootunniha meichchunniha, gerechch unniha) |  |
|  | ቀይ ስጋ (የበሬ፤ የፍየል፤ የበግ) |  |
| 516 | **Chicken meet** |  |
|  | Lukkichchu maala |  |
|  | የዶሮ ሰጋ |  |
| 517 | **Fish** |  |
|  | Qulxume |  |
|  | አሳ |  |
| 518 | **Internal organ (liver, kidney, heart)** |  |
|  | Giddoodi mannimma gaamo maalal fale(kulalitite, wodana) |  |
|  | የውሰጥ የሰውነት ክፍል ሥጋ (ጉበት፣ ኩላሊት፣ልብ) |  |
| 519 | **Coffe or tea** |  |
|  | Shaenna buna |  |
|  | ሻይ ወይም ቡና |  |
| 520 | **Soft drink** |  |
|  | Shaffado ago |  |
|  | ለስላሳ መጠጦች |  |
| 521 | **Any alchol** |  |
|  | Kajjado ago |  |
|  | ዐልኮል መጠጦች |  |
| 522 | **Others specify** |  |
|  | Wole kunni assi |  |
|  | ሌላ ካለ ግለፅ/ጪ |  |

| **Part VI Supplementation question** | |  |  |  |
| --- | --- | --- | --- | --- |
| **Kifile 6 Ledishu sagale xa’mo** | |  |  |  |
| **ክፍል 6፤ ተጨማሪ ምግብ ስለመውሰድ የሚገልጽ መጠይቅ** | |  |  |  |
|  | **Question** |  |  | **Code** |
| 601 | **Do you feed the child any foods made with oil, fat or butter?** |  |  |  |
|  | Zayite, buuronna,coomu sagale saga’lano? | 0. [_] No | 0.Disagalino[__] | 0[__]አይደለም |
|  | ልጅሸን ዘይት፤ ጮማ ወይም ቅቤ ያለበትን ምግብ ትመግቢዋለሽ | 1. [_] Yes | 1.Sagalino[__] | 1[__]አዎን |
|  | **If yes for question number 601 do you feed every day** |  |  |  |
| 602 | Barrunni daaimu me’e marro saga’lano | 0. [_] No | 0.Disagalino[__] | 0[__]አይደለም |
| ዝለል Skip | ከመገብሽው በየቀኑ ትመግቢዋለሽ | 1. [_] Yes | 1. Sagalino[__] | 1 [__]አዎን |
| 603 | **Has your child ever received Iron supplementation** |  |  |  |
|  | Daaimu ayirenete lattinota ledishu sagale afiranno? | 0. [_] No | 0. Disagalino[__] | 0[__]አይደለም |
|  | የደም መሙያ አንክብል ወሰዶ ያውቃል? |  | 1. Sagalino[__] | 1[__]አዎን |
|  | **If yes for question number 603 Why he or she received?** |  |  |  |
| 604 | 19kki xamo saga’lanoha ikiro Mayirra sagalanno? |  |  |  |
| ዝለል skip | የደም መሙያ አንክብል ከወሰደ ለምንድን ነው የወሰደው |  |  |  |
|  | **From where do you get?** |  |  |  |
| 605 | Maminni afidhinonni? | 1.[_] From health institution | 1[_]Fayyimateuurishinni | 1 [_]ከጤና ተቋም |
| ዝለል skip | የደም መሙያ አንክብል ከየት ነው የተሰጠው? | 2.[_] Food aid program | 2[_]Sagallte qixawonni | 2 [_]ከምግብ ኘሮግራም |
|  |  | 3.[_]Pharmacy | 3[_]Farmasetenni | 3 [_]ፋርማሲ |
|  |  | 4. [_]Other specify | 4[_]Wolekuli | 4 [_]ሌላጥቀስ |
|  |  |  |  |  |
| 606  ዝለል  Skip | **Does the child taking now?** |  |  |  |
|  | Qaaqqu xaano mundee abbitawoota xagicho adhayno? | 0. [_] No | 0.Disagalino[__] | 0[__]አይወስድም |
|  | ህፃኑ አሁንም የደም መሙያ እንክብል አየወሰደ ነው? | 1. [_] Yes | 1.Sagalino[__] | 1[__]አዎ |
| 607  ዝለል  Skip | **For how long he /she take supplementation?** |  |  |  |
|  | Ladishshu sagale mageshi geeeshsha sagalonno? |  |  |  |
|  | የደም መሙያ ዕንክብል ከወሰደ ለምን ያህል ጊዜ ወሰደ በወራት ጥቀሽ/ስ? |  |  |  |
| 608 | **Has your child ever eat iron fortified food** |  |  |  |
|  | Daaimikki Ayiirenetenni kaajjinshoonni sagle sagalanno? | 0. [_] No | 0. Disagalino[__] | 0[__]አይወስድም |
|  | ህፃንሸ ደምን በሚሞላ የብረት ንጥረ ነገር የበለፀገ ምግብ በልቶ ያውቃል? | 1. [_] Yes | 1. Sagalino[__] | 1[__]አዎ |
| 609  ዝለል  Skip | **If yes for question number 608** |  |  |  |
|  | **From where do you get** |  |  |  |
|  | Hiikiinni afiranno? | 1.[_] From health institution | 1[_]Fayyimateuurishinni | 1[_]ጤናተቋም |
|  | ከበላ ከየት ነው ያገኘሽው? | 2.[_] Food aid program | 2[_]Sagallte qixawonni | 2[_]የምግብ ኘሮ ግራም |
|  |  | 3.[_]Pharmacy | 3[_]Farmasetenni | 3[_]ፋርማሲ |
|  |  | 4. [_]Other specify | 4[_]Wolekuli | 5[_]ሌላ ካለ (ዘርዝር/ሪ) |
| 610  ዝለል  Skip | **Is the child taking now?** |  |  |  |
|  | Mamoote saga’lanno? | 0. [_] No | 0[_]Disagalino | 0[_]አይደለም |
|  | አሁንም ህጻኑ አየተመገበ ነው( ዕየተመገበ ከሆነ ዐሳዪኝ)? | 1. [_] Yes | 1[_]Sagalino | 1[_]አዎን |
|  | What was the food? |  |  |  |
| 611  ዝለል  Skip | Sagale maati? |  |  |  |
|  | የሚወስደው የበለጸገ ምግብ ምን ነበር( ያሳየችህን የምግብ ዐይነት ፃፍ?) |  |  |  |
| 612  ዝለል  Skip | **For how long she/he take?** |  |  |  |
|  | Mageeshshi geeshe saga’lano? |  |  |  |
|  | የበለጸገ ምግቡን ለምን ያህል ጊዜ ወሰደ(ወሰደች) በወራት ጥቀሽ/ስ? |  |  |  |
| 613 | **Has the child ever been given a Vit A capsule?** |  |  |  |
|  | Daaimu vitaamine A kinine afire egennino? | 0. [_] No | 0. [_]Disagalino | 0. [_]አያውቅም |
|  | ህፃኑ የሻይታሚን A እንክብል ወሰዶ ያውቃል? | 1. [_] Yes | 1. [_]Sagalino | 1. [_] አዎ |
| 614  ዝለል  Skip | I**f yes for question no 613 how often did he/she get** |  |  |  |
|  | kki xa’mo afirinnoha ikkiro ,me’e marro afirino? |  |  |  |
|  | ቫይታሚን A ከወሰደ በአመት ምን ያህል ግዜ ያገኛል? |  |  |  |
| 615 | **Since you were pregnant Have you taken any Iron supplements?** |  |  |  |
|  | Godowii heedhe ayiirenete lattino sagale afiroota? | 0. [_] No | 0. [_]Disagalino | 0. [_]አያውቅም |
|  | እርጉዝ ሆነሸ ደም የሚሞላ እንክብል ወስደሽ ታውቂያለሸ? | 1. [_] Yes | 1. [_]Sagalino | 1. [_] አዎ |
| 616  ዝለል  Skip | **If yes for question number 21 for how long did you taken the supplement** |  |  |  |
|  | 21kki xa’mo afirootana ikkiro mageeshi geeshsha afiroota? | 1. [_] less than month | 1. [_] 1 agani woro | 1 [_] ከአንድ ወር በታች |
|  | ደም የሚሞላ ዕንክብል ከወሰድሸ ለምን ያህል ጊዜ ነው የወሰድሸው? | 2. [_] 1 up 2 month | 2. [_] 1-2 agani geesha | 2. [_] 1-2ወር |
|  |  | 3. [_] 2up3 | 3. [_] 2-3 agani geesha | 3. [_] ከ2-3 ወር |
|  |  | 4. [_] more than 3 month | 4. [_] 3 apani ale | 4. [_] ከ3 ወር በላይ |
|  |  | 5. [_] for 6 month  6. [_] Do not know | 5. [_] 6 adani geesha | 5. [_]ለ6ወር |
|  |  |  | 6. [_] Diafooma | 6. [_]ዐላውቅም |
|  |  |  |  |  |

| **Part 7. Baby health question** | |  |  |  |
| --- | --- | --- | --- | --- |
| **Kifile 7 Daaimu fayimu xa’mu** | |  |  |  |
| **ክፍል 7 የህፃኑን ጤና የሚገልፅ መጠይቅ** | |  |  |  |
| **Now I am going to ask you questions which are related to your baby’s health** | | |  |  |
| Xa xa’meemohehu daa’mu fayima ledo xedooshu noo xa’mooti | | |  |  |
| ዐሁን የህጻኑን ዐጠቃላይ የጤና ሑኔታ የሚገልጽ ጥያቄ ዕጠይቅሻለሁ | |  |  |  |
|  | **QUESTION** |  | **CODE** |  |
| 701 | **Does the baby sleep in your bed?** |  |  |  |
|  | Daaimu ate daalasira goxanno? | 0. [_] No | 0. [_]Dee’ni | 0. [_]አይ |
|  | ህፃኑ ከአንቺ ጋር ነው የሚተኛው | 1. [_] Yes | 1. [_]Eewa | 1. [_]አዎ |
| 702 | **Do you use bed net to your child?** |  |  |  |
|  | Daaimu goxenno woyite agobere horonsiranno? | 0. [_] No | 0. [_]Dee’ni | 0. [_]አይ |
|  | ህፃኑ በአጎበር ተሸፍኖ ነው የሚተኛው | 1. [_] Yes | 1. [_]Eewa | 1. [_]አዎ |
| **703** | **Does the child vaccinated ?** |  |  |  |
|  | Daaimu kittibaate garunni gudino? | 0. [_] No | 0. [_]Dee’ni | 0. [_]አይ |
|  | ህፃኑ ክትባት ወስዷል? | 1. [_] Yes | 1. [_]Eewa | 1. [_]አዎ |
| **704** | **Does the child had vaccine card?** |  |  |  |
|  | Kittibaate gudino kaarde noosi? | 0. [_] No | 0. [_]Dee’ni | 0. [_]አይ |
|  | ህፃኑ የክትባት ካርድ አለው? | 1. [_] Yes | 1. [_]Eewa | 1. [_]አዎ |
|  | **Could you show me vaccine card (please find which vaccine the child take from the card?)** |  |  |  |
| 705 | BCG kitibaate dasirino anga lea hasireemona lao? | [_]BCG? | [_]BCG? | [_]የሳንባ ምች |
|  | Dasirinohu qinniticho angara dasahootic? | [_]Polio 0? | [_]Polio 0? | [_]የህፃናት ልምሻ 0 |
|  |  | [_]Polio1? | [_]Polio1? | [_]የህፃናት ልምሻ 1 |
|  | የክትባቱን ካ ርድ ታሳዩኛለሸ (በክትባት ካርድ ላይ የወሰደውን X ያልወሰደውን 0 አድርግ/ጊ ) | [_]Polio2? | [_]Polio2? | [_]የህፃናት ልምሻ 2 |
|  |  | [_]Polio3? | [_]Polio3? | [_]የህፃናት ልምሻ 3 |
|  |  | [_]DPT- 1 | [_]DPT- 1 | [_]ዲፒቲ 1 |
|  |  | [_]DPT- 2 | [_]DPT- 2 | [_]ዲፒቲ 2 |
|  |  | [_]DPT- 3 | [_]DPT- 3 | [_]ዲፒቲ 3 |
|  |  | [_]PCV1 | [_]PCV1 | [_]ፒሲ ቪ1 |
|  |  | [_]PCV2? | [_]PCV2? | [_]ፒሲ ቪ 2 |
|  |  | [_]PCV3? | [_]PCV3? | [_]ፒሲቪ3 |
|  |  | [_]Rota1? | [_]Rota1? | [_]ሮታ 1 |
|  |  | [_]Rota2? | [_]Rota2? | [_]ሮታ 2 |
|  |  | [_]Measealse | [_]Measealse | [_]ኩፍኝ |
| 706 | **Can I see the BCG scar please?** |  |  |  |
|  | BCG kitibaate dasirino anga lea hasireemona lao? | 1.[_] BCG – lesion seen: | 1.[_] BCG-bassa leellanno | 1 የክትባቱ ጠባሳ ይታያል |
|  | Dasirinohu qinniticho angara dasahootic | 2.[_] BCG – lesion not seen | 2.[_] BCG-bassu dileellanno | 2 የክትባቱ ጠባሳ አይታይም |
|  | የሳንባ ነቀርሳ ክትባት ቦታውን ማየት እቸላለው? |  |  |  |

| **Part 7.1 Morbidity**, | |  |  |  |
| --- | --- | --- | --- | --- |
| **Diarrhea episode two weeks recall** | |  |  |  |
| **ክፍል 7.1:** ባለፉት 2 ሳምንታት ስለ ህፃኑ የተቅማጥ ህመም መጠይቅ | |  |  |  |
|  | **QUESTION** |  | **Code** |  |
| 707 | **During the last two weeks that ended yesterday morning, did the child have diarrhea?** |  |  |  |
|  | Sau leme lamalanni henafe bero soodo geeshsha deuu malaati noosi? | 0. [_] No | 0. [_]Dinosi | 0. [_]አይደለም |
|  | ባለፉት 2 ሳምንታት ህፃኑ የተቅማጥ (የሆድ) በሸታ አሞት ነበር? | 1. [_] Yes ↓ | 1. [_]Noosi | 1. [_]አዎ |
| 708  ዝለል  Skip | **Did the child pass any watery stools?** |  |  |  |
|  | Waa lawanno deiinoosi? | 0. [_] No | 0. [_]Dinosi | 0. [_]አይደለም |
|  | ህፃኑ ውሀ የሚመስል ተቅምጥ አሰቀምጦታል | 1. [_] Yes↓ | 1. [_]Noosi | 1. [_]አዎ |
| 709  ዝለል  Skip | **The day had most loose or watery stools, how many loose or watery stools did pass?** |  |  |  |
|  | Wayi gedee deoo deeanni hoosino barri no? |  |  | |
|  | ብዙ ባሰቀመጠው ቀን ምን ያህል ጊዜ አሰቀመጠው? |  |  |  |
| 710  ዝለል  Skip | **Did any of the stools contain blood?** |  |  |  |
|  | Daaimu sagari aana mundee karsantewo? | 0. [_] No | 0. [_]Dinosi | 0. [_]አይደለም |
|  | በተቅማጡ ውስጥ ደም ታይቷል | 1. [_] Yes ↓ | 1. [_]Noosi | 1. [_]አዎ |
| 711  ዝለል  Skip | **Were the stools of different consistency than before fell ill with diarrhea?** |  |  |  |
|  | Deeiishanno yannara wole yanawiinni baxxitino xisso maciishshenno? | 0. [_] No | 0. [_]Dinosi | 0. [_]አይደለም |
|  | ሲያሰቀምጠው ህመም ይሰማው ነበር | 1. [_] Yes ↓ | 1. [_]Noosi | 1. [_]አዎ |
| 712  ዝለል  Skip | **Did the illness interfere with ability to drink or eat?** |  |  |  |
|  | Sagalanno woyiitena wala aganno wayite xisso maciishantennosi? | 0. [_] No | 0. [_]Dinosi | 0. [_]አይደለም |
|  | ህመሙ ምግብ እንዳይበላ ያደርገው ነበር | 1. [_] Yes ↓ | 1. [_]Noosi | 1. [_]አዎ |
| 713  ዝለል  Skip | **Did you seek treatment for?** |  |  |  |
|  | Moyyessate wonaaloo? | 0. [_] No | 0. [_]Dinosi | 0. [_]አይደለም |
|  | ለህመሙ የህክምና እርዳታ ፈልገሸ ነበር? | 1. [_] Yes ↓ | 1. [_]Noosi | 1. [_]አዎን |
| 714  ዝለል  Skip | **Was the child admitted to a hospital?** |  |  |  |
|  | Daaimu hospitalete goxino? | 0. [_] No | 0. [_]Dinosi | 0. [_]አይደለም |
|  | ለህመሙ ሆሰፒታል ተኝቶ ነበር? | 1. [_] Yes ↓ | 1. [_]Noosi | 1. [_]አዎን |
| 715  ዝለል  Skip | **How many days did the diarrhea last?** |  |  |  |
|  | Deuu meu barri geisha keeshino? |  |  | |
|  | ተቅማጡ ለምን ያህል ጊዜ ቆየበት/ባት(በቀናትጥቀስ/ሽ) |  |  |  |
| 716  ዝለል  Skip | **During this period of illness you have described, did you change the way you were feeding your child in any way?** |  |  |  |
|  | Dhibbu yannara hitto garinnit sagale itisatahu | 1. [_] More often | 1[_] albininniraha raha | [_]1. ከበፊቱ ቶሎ ቶሎ |
|  | በህመሙ ጊዜ በምን አይነት መልኩ ነበር የምትመግቢው? | 2. [_] less seldom than before the illness started. | 2[_]Albinni asishi | [_]2. ከበፊቱ ቀንሼ |
|  |  | 3. [_] Did not change feeding frequency | 3[_]Albinni Soorro dino | [_]3.ከበፊቱ ለውጥ የለውም |

| **Part 7.2. Malaria 3 month recall** | |  |  |  |
| --- | --- | --- | --- | --- |
| **kifille 7.2. 3Aganni mereero sheekerete xisso** | |  |  |  |
| ክፍል7.2.ላለፉት 3 ወር ውስጥ ስለ ህፃኑ የወባ ህመም ሁኔታ የሚያመላክት መጠይቅ | |  |  |  |
|  |  |  |  |  |
|  | **QUESTION** |  | **CODE** | **መለያ** |
| 717 | **During the last three months that ended yesterday morning, did the child have fever?** |  |  |  |
|  | Sau 3 aganninni kayise bero soodo geshsha daaimu biso iibbabbino | 0. [_] No | 0. [_]Dinosi | 0.[_]አይደለም |
|  | ላለፉት 3 ወራት ልጅሸ ትኩሳት አሞት ያውቃል | 1. [_] Yes | 1. [_]Noosi | 1. [_]አዎን |
| 718  ዝለል  Skip | **Did you brought the child to health institute** |  |  |  |
|  | kki xa’mo “iibbabinno” ikkituro daaimu me’e marr iibbabbino? |  |  | |
|  | ትኩሳት ካመመው ለምን ያህል ጊዜ ደጋግሞ ዐመመው? |  |  |  |
| 719  ዝለል  Skip | **Did you brought the child to health institute** |  |  |  |
|  | Daaimakki fayyimmate uurrinsha massooto | 0. [_] No | 0. [_]Dinosi | 0.[_]አይደለም |
|  | ሀኪም ቤት ወስደሽው ነበር? | 1. [_] Yes | 1. [_]Noosi | 1. [_]አዎን |
| 720  ዝለል  Skip | **If yes what was the illness** |  |  |  |
|  | Massootthoa ikkiro xissosi dani maati |  |  | |
|  | ከወሰድሸው በሸታው ምን ነበር |  |  |  |
| 721  ዝለል  Skip | **During this period of illness you have described, did you change the way you were feeding your child in any way?** |  |  |  |
|  | Daaima xissosi sagale saga’lannoki pedenna agannokki gede assitinosi? | 0. [_] No | 0. [_]Dinosi | 0.[_]አይደለም |
|  | ህፃኑን በሸታው ከመብላት ከመጠጣት ከልክሎት ነበር? | 1. [_] Yes | 1. [_]Noosi | 1. [_]አዎን |
| 722  ዝለል  Skip | **Was the child admitted to a hospital for the illness?** |  |  |  |
|  | Daaimu xissame hospitalete aoxino? | 0. [_] No | 0. [_]Dinosi | 0.[_]አይደለም |
|  | ህፃኑ ባመመው ትኩሳት ምክንያት ሆሰፒታል ተኝቶ ነበር | 1. [_] Yes | 1. [_]Noosi | 1. [_]አዎን |
| 723  ዝለል  Skip | **During the period of illness did you feed your baby more often, more seldom than or just as often as before the illeness started?** |  |  |  |
|  | Saggala hiitto daagonni Soorritta | 1. [_] More often | 1[_]Albininniraha raha | 1[_]ከበፊቱ ቶሎ ቶሎ |
|  | በህመሙ ጊዜ አመጋገብ እንዴት ባለ መንገድ ቀየርሸ | 2. [_] More seldom than before the illness started | 2[_]Albinni asishi | 2[_]ከበፊቱ ቀንሼ |
|  |  | 3. [_] Did not change feeding frequency. | 3[_]Albinni Soorro dino | 3[_]ከበፊቱ ለውጥ የለውም |

| **Part 7.3: Pneumonia 2 Week recall** | |  |  |  |
| --- | --- | --- | --- | --- |
| **Kifille 7.3: Lemala mereero saambu michche xisso** | |  |  |  |
| **ክፍል 7.3 ላለፉት 2 ሳምንታት የሳንባ ምች ህመምን የሚያመላክት መጠይቅ** | |  |  |  |
|  |  |  |  |  |
|  | **QUESTION** |  | **CODE** |  |
| **724** | **During the last two weeks that ended yesterday morning, did the child have cough?** |  |  |  |
|  | Sau lame lemale kayisse be’ro soodo geeshsha, Daaimu Buusano Afirino? | 0. [_]No | 0. [_]Dinosi | 0. [_]አይደለም |
|  | ባለፉት 2 ሳምንታት ህፃኑ ሳል አሞት ነበር ? | 1. [_]Yes | 1. [_]Noosi | 1. [_]አዎን |
|  | **During the last two weeks that ended yesterday morning, did the child have fast or difficult breathing?** |  |  |  |
| 725  ዝለል  Skip | Sau lemalaa kayiise be’ro soodo geeshsha. Daaimu rahe rare fo’lanno woy foo’late rakkatanno? | 0. [_]No | 0. [_]Dinosi | 0. [_]አይደለም |
|  | ላለፋት 2 ሳምንታት እሰከ ትላንት ጠዋት ህፃኑ ትንፋሽ ያጥረው ነበር ? | 1. [_]Yes | 1. [_]Noosi | 1. [_]አዎን |
| 726  ዝለል  Skip | **Did the illness interfere with the child ability to drink or eat?** |  |  |  |
|  | Xisso ittennona agannoki gede rakkatisannosi? | 0. [_]No | 0. [_]Dinosi | 0. [_]አይደለም |
|  | የህመሙ ሁኔታ መብላትና መጠጣት ዕንዲያስቸግረው ዐድርጎበት ነበር? | 1. [_]Yes | 1. [_]Noosi | 1. [_]አዎን |
| 727  ዝለል  Skip | **Was the child admitted to a hospital for the illness?** |  |  |  |
|  | Daaimu xissame hospiitalete goxinno? | 0. [_]No | 0. [_]Dinosi | 0. [_]አይደለም |
|  | ህፃኑ በህመሙ ምክንያት ሆሰፒታል ተኝቶ ነበር? | 1. [_]Yes | 1. [_]Noosi | 1. [_]አዎን |
| 728  ዝለል  Skip | **During the period of illness did you feed your baby more often, more seldom than or just as often as before the illness started?** |  |  |  |
|  | Konni dhibbinni daaimakki hiittoonniti itisaffahu | 1. [_] More often | 1. A lbininniraha raha | 1[_] ከበፊቱ ቶሎ ቶሎ |
|  | በዚህ ህመም ህፃንሽን አንዴት ነበር የምትመ ግቢው? | 2. [_] More seldom than before the illness started | 2. Albinni asishi | 2[_] ከበፊቱ ቀንሼ |
|  |  | 3. [_] Did not change feeding frequency. | 3. Albinni Soorro dino | 3[_]ከበፊቱ ለውጥ የለውም |

| **Part 7.4:Hospitalizations** | |  |  |  |
| --- | --- | --- | --- | --- |
| **Kiffile 7.4:Hospiitaalete goxinno yanna** | |  |  |  |
| **ክፍል 7.4፡የህፃኑ ሆስፒታል የመተኛት ሁኔታ** | |  |  |  |
|  | **QUESTION** |  |  | **CODE** |
| **729** | **Since birth has ever been admitted to hospital?** |  |  |  |
|  | Daaimu ilami yanaa henafe hospiitalete goxe egennino | 0. [_] No | 0. [_]Dinosi | 0. [_]አይደለም |
|  | ህፃኑ ከተወለደ ጀምሮ ሆስፒታል ተኝቶ ያውቃል? | 1. [_] Yes ↓ | 1. [_]Noosi | 1. [_]አዎን |
| 730  ዝለል  Skip | **How many times has been admitted to hospital?** |  |  |  |
|  | Hospitaalete me’e marro goxino? |  |  |  |
|  | ሆስፒታል ለምን ያህል ጊዜ ደጋግሞ ተኝቶዐል? |  |  |  |
| 731  ዝለል  Skip | **What was the maximum days admited t hospital** |  |  |  |
|  | Barra ikkanno hospitalete seeda yanna goxinohu mee barra ikkanno |  |  |  |
|  | በሆሰቲታል ለረጅም ጊዜ የተኛው ምን ያህል ቀን ነው? |  |  |  |
|  | **What was the reason was in the hospital each time:** |  |  |  |
| 732  ዝለል | Hospiitaalete mayi xinsoonnit | 1. [_] 1^st^ | 1. [_] 1^st^ | 1. [_] 1^st^ |
|  | ሆስፒታል የተኛበት ምክንያት ምን ነበር ለእያንደንዱ ከሚከተሉት ምርጫ አሰቀምጥ/ጭ | 2. [_] 2^nd^ | 2. [_] 2^nd^ | 2. [_] 2^nd^ |
|  | 1. Malaria/ወባ/wobahoni | 3. [_] 3^rd^ | 3. [_] 3^rd^ | 3. [_] 3^rd^ |
|  | 2. Pnemonia/የሳንባምች/sanbamich | 4. [_] 4^th^ | 4. [_] 4^th^ | 4. [_] 4^th^ |
|  | 3. gastrointeritis/ተቅማጥ/gadawa gameetini | 5. [_] 5^th^ | 5. [_] 5^th^ | 5. [_] 5^th^ |
|  | 4. any respiratory disease/ማንኛውም የመተንፈሻ አካል በሸታ/ayee foolate bisishibaah | 6. [_] 6^th^ | 6. [_] 6^th^ | 6. [_] 6^th^ |
|  | 5. ሌለ ጥቀሰ/woleri kuli | 7. [_] 7^th^ | 7. [_] 7^th^ | 7. [_] 7^th^ |
|  |  | 8. [_] 8^th^ | 8. [_] 8^th^ | 8. [_] 8^th^ |

| **Part 8.1: Mother knowledge on Anemia** | |  |  | | |  |
| --- | --- | --- | --- | --- | --- | --- |
| **Kiffille 8.1 Aneemiyu (mundeete anje) aana amate no huwato** | |  |  | | |  |
| ክፍል 8.1. በደም ማነስ ህመም ላይ የናትየው ዕውቀት | |  |  | | |  |
|  |  |  |  | | |  |
| **No** | **Question** |  |  | | | **Remark** |
|  | **Did you ever heard about anemia** |  |  | | |  |
| 801 | Aneemiyu xisso maatiro macciishite egennota ? | 0.[_] No | 0.[_] Dee’ni | | | 0. [_]አይደለም |
|  | ስለ ደም ማነስ ሰምተሸ ታውቂያለሽ? | 1.[_] Yes | 1.[_] Eewa | | | 1. [_]አዎን |
| 802  Skip  ዝለል | **If yes for question number what does it mean?** |  |  | | |  |
|  | Aneemiya yaa mayaate? |  |  |  |  |  |
|  | ሰምተሸ ካወቅሸ ምን ማለት ነው? |  |  |  |  |  |
| 803  Skip  ዝለል | **How can anemia are prevented** |  |  | | |  |
|  | Aneemiyu xisso hiitoonni gargadhinanni? |  |  |  |  |  |
|  | የደም ማነስ እንዴት መከላከል ይቻላል? |  |  |  |  |  |
| 804  Skip  ዝለል | **Do you think anemia is sever problem** |  |  | | |  |
|  | Aneemiyu lowo geeshsha gawajjanota huwatootta? | 0.[_] No | 0.[_] Dee’ni | | | 0. [_]አይደለም |
|  | የደም ማነሰ ከባድ የጤና ቸግር ነው ብለሸ ታስቢያለሸ | 1.[_] Yes | 1.[_] Eewa | | | 1. [_]አዎን |
| 805  Skip  ዝለል | **If no what is your reason** |  |  | |  | |
|  | Huwatootta ikkiha ikkiro, korkaatu maati? |  |  |  |  |  |
|  | ከባድ ነው ብለሸ ካልሸ ምክንያትሽ ምንድን ነው? |  |  |  |  |  |
| 806  Skip  ዝለል | **What action you will did if you know your child is anemic** |  |  |  | | |
|  | Daaimikki aneemiyu xissonni amadamiro adhatta qaafo maati? |  |  |  |  |  |
|  | ህፃንሽ በደም ማነስ ቢታመም ምን ታደርጊያለሸ? |  |  |  |  |  |

| **Part 8.2 Mother knowledge on iron rich food** | |  |  | |  |
| --- | --- | --- | --- | --- | --- |
| **Kiffile 8.2 ama/lossaancho ayiirene guuttino sagale aana nose huwato** | | |  | |  |
| **ክፍል 8.2.የናትየው ዕውቀት በብረት የበለፀጉ ምግቦች ላይ** | |  |  | |  |
| **No** | **Question** |  |  | |  |
| 807 | **Have you ever heard about iron rich food if yes go to question number 808** |  |  | |  |
|  | 72^kki^ Kiiro aana ayyirene guutino sagale macciishootta? | 0.[_] No | 0.[_] Dee’ni | | 0. [_]አይደለም |
|  | በብረት ስለበለፀጉ ምግቦች ሰምተሽ ታውቂያለሸ? | 1.[_] Yes | 1.[_] Eewa | | 1. [_]አዎን |
| 808  Skip  ዝለል | **From where did you hear about iron rich food?** |  |  | |  |
|  | Ayirene guuttino sagale mamminni macciishitta? | 1.[_]Health staff | 1[_] Fayimmate loosasinewinni | | [_]1. ከጤና ባለሞያ |
|  | ካወቅሸ ከየት ነው የሰማሽው? | 2.[_]Radio | 2[_] Raddonetenni | | [_]2. ከብዙሀን መገናኛ |
|  |  | 3.[_]NGO | 3[_] Mangistawe ikkitinokki uurrinshanni. | | [_]3.መንግሰታዊ ካለሆኑ ድርጅቶች |
|  |  | 4.[_]Family | 4[_] Maate’yawiinni | | [_]4.ከቤተሰብ |
|  |  | 5.[_]Don’t know | 5[_] Diafoomma | | [_]5.አላውቅም |
|  |  | 6. [_]Other (specify) | 6[_] Wolewiinni (xawisi) | | [_]6.ሌላ ግለፅ |
| 809  Skip  ዝለል | **Can you list some of iron rich food?** |  | | | |
|  | Ayirene guuttino sagale hiikkoreetiro kulattaera dandaatta? |  |  |  |  |
|  | በብረት ንጥረ ነገር የበለፀጉ ምግቦችን ጥቀሺ? |  |  |  |  |
| 810  Skip  ዝለል | **When taken during meals, certain foods help the body absorb and use iron. What are those foods?** |  | | | |
|  | Hurbaaxinnemmo yannara bissinke ayirene raha horonsi’ranno gede asisitanno sagale hikkoreeti? |  |  |  |  |
|  | አንዳንድ ምግቦች ከምግብ ጋር ዐብረው ሲወሰዱ የብረት ንጥረ ነገር በቀላሉ ሰውነታችን እንዲጠቀመው ይረዳሉ እነዚህ ምግቦች ምንድን ናችው? |  |  |  |  |
| 811  Skip  ዝለል | Some beverages decrease iron absorption when taken with meals. Which ones? | 1.[_]Tea | 1[_] Shae | 1[_]ሻይ | |
|  | Mite mite agatto sagalete ledo anganni woyiite ayiirene ajanno gede asitannori hiikkoreeti? | 2.[_]Coffee | 2[_] Bunu | 2[_]ቡና | |
|  | አንዳንድ መጠጦች የብረት ንጥረ ነገር ሰውነታችን ዕንዳይጠቀም ይከለክላሉ ዕነዚህ የምግብ ዐይነቶች አነማን ናቸው? | 3.[_]Other specify | 3[_] Diafoomma | 3[_]አላውቅም | |
|  |  | 4.[_]Don’t know | 4[_] Wolere (xawisi) | 4[_]ሌለ ጥቀሰ | |
| 812  Skip  ዝለል | **How good do you think it is to prepare meals with iron-rich foods such as beef, chicken or liver?** |  |  |  | |
|  | Ayirene guuttino sagale danchu garinni hiitto qixxesinanniro afootta? Lawishaho bootu maala,lukkichu maalanna salto lawinore. | 1.[_]Not good | 1[_] Didanchaho | 1[_]ጥሩ አይደለም | |
|  | በብረት የበለፀጉ ምግቦቸን ማለትም የበሬ ስጋ የዶሮ ሰጋና ጉበት፤ ኩላሊት ከህፃናት ምግብ ጋር ቀላቅሎ መስራት ጥሩ ነው ብለሽ ታስቢያለሽ? | 2.[_]Good | 2[_] dibbuxomma. | 2[_]ጥሩ ነው | |
|  |  | 3.[_]You’re not sure | 3[_]Danchaho | 3[_]እርግጠኛ አይደለሁም | |
|  |  | 4.[_]I don’t know |  | 4[_]አላውቅም | |
| 813  Skip  ዝለል | **If it is not good. Can you tell me the reasons why it is not good?** |  | | | |
|  | Dancha ikkinokiha ikkiro:Dancha ikkinokki Korkaati maati? |  |  |  |  |
|  | ጥሩ ካልሆነ ምክንያትሸ ምንድን ነው |  |  |  |  |
| 814  Skip  ዝለል | **If it is not good. Can you tell me the reasons why it is not good?** |  | | | |
|  | Dancha ikkinokiha ikkiro:Dancha ikkinokki Korkaati maati? |  |  |  |  |
|  | ጥሩ ከሆነ ምክንያትሸ ምንድን ነው |  |  |  |  |
| 815  Skip  ዝለል | **How difficult is it for you to prepare meals with iron-rich foods?** | [_]Not difficult | [_] injaannoe | 1ከባድ አይደለም | |
|  | Dancha ikkinokiha ikkiro: Dancha ikkinokki Korkaati maati? | [_]So-so | [_] hageeshi geeshaati. | 2ከባድ ነው | |
|  | ላንቺ በብረት የበለፀጉ ምግቦችን ማዘጋጀት ምን ያሀል ከባድ ነው? | [_]Difficult | [_] Di injannoe | 3በጣም ከባድ ነው | |
| 816  Skip  ዝለል | **If difficult. Can you tell me the reasons why it is difficult?** |  | | | |
|  | Injaannohe ikkiha ikkiro: Korkaatu maati? |  |  |  |  |
|  | ከባድ ከሆነ ምክንያቱ ምንድን ነው |  |  |  |  |
| 817  Skip  ዝለል | **If it is not difficult. Can you tell me the reasons why it is difficult?** |  | | | |
|  | Injaannohe ikkiha ikkiro: Korkaatu maati? |  |  |  |  |
|  | ከባድ ካልሆነ ምክንያትሽ ምንድን ነው |  |  |  |  |

Part 9**: Household Food Insecurity Access Scale (HFIAS) Measurement:**

**Now I would like to ask you few questions regarding your household food security situation in the past four weeks.**

**ክፍል 9 ላለፉት 4 ሳምንታት የቤተሰቡን የምግብ ዋስትና የሚያሳይ መጠይቅ**

| **Rule**: We, will not give "timing" instructions and force answers.  Reported frequency in correct category according to this list:  0. No/never,  1. Sometimes: 1-2 days last month,  2. Often: 3-10 days/month,  3. Very often/usually: More than 10 days last month | | በሚከተለው ገለፃ መሰረት የዕናትየውን መልስ ዐስቀምጥ/ጪ  1ወ ይም 2 ጊዜ ላለፉት 4 ሳምንታት (ጥቂት ጊዜ)  ከ3 -10 ጊዜ ላለፉት 4 ሳምንታት (ዐንዳንድ ጊዜ)  ከ10 ጊዜ በላይ ላለፉት 4 ሳምንታት (ብዙ ጊዜ ) | | |
| --- | --- | --- | --- | --- |
| **ተ/ቑ** | **ጥያቄ (questions)** |  |  |  |
|  |  |  |  |  |
| **901** | **In the past four weeks, did you worry that your household would not have enough food?** | |  |  |
|  | Sau shoole lamalara miniki manni ikkado sagala afirewokkhura qarranten egenootta | 0. [_]No | 0. [_]Dee’ni | 0. [_]አይደለም |
|  | ባለፉት ዐራት ሳምንታት ለቤተሰብሽ በቂ ምግብ ካለመኖሩ የተነሣ ተጨንቀሽ ታውቂያለሽ | 1. [_]Yes | 1. [_]Eewa | 1. [_]አዎን |
|  | **How often did this happen in the past four weeks?** |  |  |  |
| 902  skip  ዝለል | Qarrante egendottara shoole lamala giddo mageeshshi yanna ikkanno | [_]1.Rarely (once or twice in the pastfour weeks) | [_]1.Harancho hanna | [_]1.ጥቂት ጊዜ |
|  | ባለፉት 4 ሳምንታት ለምን ያህል ጊዜ ዐጋጠመሽ | [_]2.Sometimes ( three to ten times inthe past four weeks) | [_]2.Sae sae | [_]2. ዐንዳንድ ጊዜ |
|  |  | [_]3.Often (more than ten times the past four weeks) | [_]3.Seeda yanna | [_]3. ብዙ ጊዜ |
|  | **In the past four weeks, were you or any household member not able to eat the kinds of foods you preferred because of a lack of resources?** |  |  |  |
| 903 | Sau shoole lamalara hidha hoogate kainohunni ati woy minikki maate sagala hasidhinoonni sagalete dano ita hooggine egentinoonni | 0. [_]No | 0. [_]Dee’ni | 0. [_]አይደለም |
|  | ባለፉት ዐራት ሳምንታት መግዛት ካለመቻል የተነሳ ዐንቺ ወይም ከቤተሰብሽ ዐባላት መብላት የፈለጋችሁትን የምግብ ዐይነት ሳትመገቡ ቀርታችሁ ታውቃላችሁ | 1. [_]Yes | 1. [_]Eewa | 1. [_]አዎን |
| 904  Skip  ዝለል | How often did this happen in the past four weeks? |  |  |  |
|  | Hoogginihunni mageeshshi barra ikkanno xaadenehu | [_]1.Rarely (once or twice in the pastfour weeks) | [_]1.Harancho hanna | [_]1.ጥቂት ጊዜ |
|  | ባለፉት 4 ሳምንታት ለምን ያህል ጊዜ ዐጋጠመሽ | [_]2.Sometimes ( three to ten times inthe past four weeks) | [_]2.Sae sae | [_]2. ዐንዳንድ ጊዜ |
|  |  | [_]3.Often (more than ten times the past four weeks) | [_]3.Seeda | [_]3. ብዙ ጊዜ |
|  | **In the past four weeks, did you or any household member have to eat a limited variety of foods due to a lack of resources?** |  |  |  |
| 905 | Sau shoole lamala hoongunni kainohunni at way minikki maate mitte bikka callo sagalete dana sagalitinoonni | 0. [_]No | 0. [_]Dee’ni | 0. [_]አይደለም |
|  | ባለፉት ዐራት ሳምንታት መግዛት ካለመቻል የተነሳ ዐንቺ ወይም ከቤተሰብሽ ዐባላት ውስጥ ዐንድ ዐይነት ምግብ ብቻ ተመግባችሁ ታውቃላችሁ | 1. [_]Yes | 1. [_]Eewa | 1. [_]አዎን |
| 906  Skip  ዝለል | **How often did this happen in the past four weeks?** |  |  |  |
|  | Ikkiro sau shoole lamalara mageeshshi geeshshooti | [_]1.Rarely (once or twice in the pastfour weeks) | [_]1.Harancho hanna | [_]1.ጥቂት ጊዜ |
|  | ባለፉት 4 ሳምንታት ለምን ያህል ጊዜ ዐጋጠመሽ | [_]2.Sometimes ( three to ten times inthe past four weeks) | [_]2.Sae sae | [_]2. ዐንዳንድ ጊዜ |
|  |  | [_]3.Often (more than ten times the past four weeks) | [_]3.Seeda | [_]3. ብዙ ጊዜ |
| 907 | **In the past four weeks, did you or any household member have to eat some foods that you really did not want to eat because of a lack of resources to obtain other types of food?** |  |  |  |
|  | Sau shoole lamalara hoongunni kainohunni ati woy miniki manni giddo mittu ita hasidhinayikki sagalaittineegentinooni | 0. [_]No | 0. [_]Dee’ni | 0. [_]አይደለም |
|  | ባለፉት ዐራት ሳምንታት መግዛት ካለመቻል የተነሳ ዐንቺ ወይም ከቤተሰብሽ ዐባላት መብላት የማትፈልጉትን ምግብ በልታችሁ ታውቃላችሁ | 1. [_]Yes | 1. [_]Eewa | 1. [_]አዎን |
| 908  Skip  ዝለል | **How often did this happen in the past four weeks?** |  |  |  |
|  | Kuni mageeshshi geeshshaatisau shoole lamalara ikkeuohu | [_]1.Rarely (once or twice in the pastfour weeks) | [_]1.Harancho hanna | [_]1.ጥቂት ጊዜ |
|  | ባለፉት 4 ሳምንታት ለምን ያህል ጊዜ ዐጋጠመሽ | [_]2.Sometimes ( three to ten times inthe past four weeks) | [_]2.Sae sae | [_]2. ዐንዳንድ ጊዜ |
|  |  | [_]3.Often (more than ten times the past four weeks) | [_]3.Seeda | [_]3. ብዙ ጊዜ |
|  | **In the past four weeks, did you or any household member have to eat a smaller meal than you felt youneeded because there was not enough food?** |  |  |  |
| 909 | Sau shoole lamalara atway minikki maate giddomitto ikkado sagala heera hoogatenni kainohunni shiima ikkado sagala sagalino | 0. [_]No | 0. [_]Dee’ni | 0. [_]አይደለም |
|  | ባለፉት ዐራት ሳምንታት መግዛት ካለመቻል የተነሳ ዐንቺ ወይም ከቤተሰብሽ ዐባላት መብላት ከሚገባው መጠን በታች በልታችሁ ታውቃላችሁ | 1. [_]Yes | 1. [_]Eewa | 1. [_]አዎን |
|  | **How often did this happen in the past four weeks?** |  |  |  |
| 910  Skip  ዝለል | Sagalino sau shoole lamalara mageshshi yanna geeshshati xaadinohu | [_]1.Rarely (once or twice in the pastfour weeks) | [_]1.Harancho hanna | [_]1.ጥቂት ጊዜ |
|  | ባለፉት 4 ሳምንታት ለምን ያህል ጊዜ ዐጋጠመሽ | [_]2.Sometimes ( three to ten times inthe past four weeks) | [_]2.Sae sae | [_]2. ዐንዳንድ ጊዜ |
|  |  | [_]3.Often (more than ten times the past four weeks) | [_]3.Seeda | [_]3. ብዙ ጊዜ |
|  | **In the past four weeks, did you or any other household member have to eat fewer meals in a day because there was not enough food?** |  |  |  |
| 911 | Sau shoole lamalara ikkado sagale heera hoogatenni kainohunni at woy minikkki maate giddo mittu barru giddo sagala noosi yanna sagalinokki yanna no | 0. [_]No | 0. [_]Dee’ni | 0. [_]አይደለም |
|  | ባለፉት 4 ሳምንታት በቂ ምግብ ካለመኖሩ የተነሳ ዐንቺ/ከቤተሰብሽ መካከል ምብላት ባለበት ሰዐት ያልተመገበ ዐለ | 1. [_]Yes | 1. [_]Eewa | 1. [_]አዎን |
| 912  Skip  ዝለል | **How often did this happen in the past four weeks?** |  |  |  |
|  | Sau lamalara mee yanna geeshshaati xaadinonehu | [_]1.Rarely (once or twice in the pastfour weeks) | [_]1.Harancho hanna | [_]1.ጥቂት ጊዜ |
|  | ባለፉት 4 ሳምንታት ለምን ያህል ጊዜ ዐጋጠመሽ | [_]2.Sometimes ( three to ten times inthe past four weeks) | [_]2.Sae sae | [_]2.ዐንዳንድ ጊዜ |
|  |  | [_]3.Often (more than ten times the past four weeks) | [_]3.Seeda | [_]3. ብዙ ጊዜ |
|  | **In the past four weeks, was there ever no food to eat of any kind in your household because of lack of resources to get food?** |  |  |  |
| 913 | Sau lamalara anjetennikainohunni sagale minigiddo hooge epentewo | 0. [_]No | 0. [_]Dee’ni | 0. [_]አይደለም |
|  | ባለፉት 4 ሳምንታት መግዛት ካለመቻል የተንሳ ማንኛዉም ዐይነት ምግብ ከቤትሽ ጠፍቶ ያውቃል | 1. [_]Yes | 1. [_]Eewa | 1. [_]አዎን |
|  | **How often did this happen in the past four weeks?** |  |  |  |
| 914 | Ikkiromageshshi yanna geeshshaati | [_]1.Rarely (once or twice in the pastfour weeks) | [_]1.Harancho hanna | [_]1.ጥቂት ጊዜ |
| ዝለል | ባለፉት 4 ሳምንታት ለምን ያህል ጊዜ ዐጋጠመሽ | [_]2.Sometimes ( three to ten times inthe past four weeks) | [_]2.Sae sae | [_]2. ዐንዳንድ ጊዜ |
|  |  | [_]3.Often (more than ten times the past four weeks) | [_]3.Seeda | [_]3. ብዙ ጊዜ |
| 915 | **In the past four weeks, did you or any household member go to sleep at night hungry because there was not enough food?** |  |  |  |
|  | Sau shoole lamalara ati woy minnikki maate giddo mittu sagalete anjoninni kainohunni hudiisannassi gaxinohu no | 0. [_]No | 0. [_]Dee’ni | 0. [_]አይደለም |
|  | ባለፉት 4 ሳምንታት ዐንቺ/ከቤተሰብሽ መካከል ከምግብ ዕጥረት የተነሳ ዕየራበው ሳይበላ የተኛ ዐለ | 1. [_]Yes | 1. [_]Eewa | 1. [_]አዎን |
|  |  |  |  |  |
| 916  Skip  ዝለል | **How often did this happen in the past four weeks?** |  |  |  |
|  | Ikkiro mageeshshi yanna geeshshaati sau shoole lamalara | [_]1.Rarely (once or twice in the pastfour weeks) | [_]1.Harancho hanna | [_]1.ጥቂት ጊዜ |
|  | ባለፉት 4 ሳምንታት ለምን ያህል ጊዜ ዐጋጠመሽ | [_]2.Sometimes ( three to ten times inthe past four weeks) | [_]2.Sae sae | [_]2. ዐንዳንድ ጊዜ |
|  |  | [_]3.Often (more than ten times the past four weeks) | [_]3.Seeda | [_]3. ብዙ ጊዜ |
| 917 | **In the past four weeks, did you or any household member go a whole day and night without eating anything because there was not enough food?** |  |  |  |
|  | Sau shoole lamalara at way minikki maate giddo mittu sagalete anjenni kainohunni sagalikkinni 24 saate keeshshinohu no | 0. [_]No | 0. [_]Dee’ni | 0. [_]አይደለም |
|  | ባለፉት 4 ሳምንታት ዐንቺ/ከቤተሰብሽ መካከል ከምግብ ዕጥረት የተነሳ ሳይመገብ 24 ሰዐት የቆየ ዐለ | 1. [_]Yes | 1. [_]Eewa | 1. [_]አዎን |
|  |  |  |  |  |
| 918  Skip  ዝለል | **How often did this happen in the past four weeks?** |  |  |  |
|  | Ikkiro mageeshshi yanna geeshshaati kalaqaminohu | [_]1.Rarely (once or twice in the pastfour weeks) | [_]1.Harancho hanna | [_]1.ጥቂት ጊዜ |
|  | ባለፉት 4 ሳምንታት ለምን ያህል ጊዜ ነው ያጋጠመሽ | [_]2.Sometimes ( three to ten times inthe past four weeks) | [_]2.Sae sae | [_]2. ዐንዳንድ ጊዜ |
|  |  | [_]3.Often (more than ten times the past four weeks) | [_]3.Seeda | [_]3. ብዙ ጊዜ |
| 919 | **Have you received any food support in the past month?** |  |  |  |
|  | Sai aganira ayita sagalete kaalo adhootta | 0. [_]No | 0. [_]Dee’ni | 0. [_]አይደለም |
|  | ባለፈው ወር ማንኛውም የምግብ ዕርዳታ ዐግኝተሻል | 1. [_]Yes | 1. [_]Eewa | 1. [_]አዎን |
| 920 | **How do you cope at times when you are running out of food in the house?** |  |  |  |
|  | Sagala mini giddo heedhu kkinni gatturo maat assahahu | 1[_].Reduce number of meals | 1[_]Adhaw sagaleaanoheayeajisheena | 1[_]የሚወሰደውን የምግብ ድግግሞሽ ቀንሳለሁ |
|  | በቤት ውስጥ የምግብ ዕጥረት ሲያጋጥምሽ ምን ዐማራጭ ነው የምትወስጂው | 2[_].Reduce meal size | 2[_]Sagalete geesha ajiplema | 2[_]የምግብ መጠን ቀንሳለሁ |
|  |  | 3[_].Borrowing | 3[_]Liqiirema | 3[_]ዕበደራለሁ |
|  |  | 4[_].Petty trade | 4[_]Hirary sagale hasileme | 4[_]የሚሸጥ ነገር ፈልጋለሁ |
|  |  | 5[_].Consume stored food (seed) | 5[_]Gootam giddo nosagale harunsitmo | 5[_]በጎተራ ያለ ምግብ ዕጠቀማለሁ |
|  |  | 6[_].Migration for labour | 6[_]Loosoho walekachcha horeema | 6[_]ለስራ ወደ ሌላ ዐካባቢ ሔዳለሁ |
|  |  | 7[_].Sell of farm tools | 7[_]Hwurayi udene hirana | 7[_]የማረሻ ዕቃዎችን ሸጣለሁ |
|  |  | 8[_].Sale charcoal/fire wood | 8[_]Ishine hirema | 8[_]ቆሻሻ ሸጣለሁ |
|  |  | 9[_].Daily labor | 9[_]Barulooso losema | 9[_]የቀን ሥራ ሰራለሁ |
|  |  | 10[_].Safety Net | 10[_]Kaalo hasireema | 10[_]ዕርዳታ ዐገኛለሁ |
|  |  | 11[_].Sell of farm animals | 11[_]Ishine adhe itema | 11[_]ከቆሻሻ ወስጄ በላለሁ |
|  |  | 12[_].Other(specify) | 12[_]Welu noose keeli | 12[_]ሌላ ካለ ጥቀስ/ሽ |
|  |  |  |  |  |
|  |  |  |  |  |

| **Section 10: Household dietary diversity** | |  |  |  |
| --- | --- | --- | --- | --- |
| **Kiffile 10. Mini mate sagalete danisagala** | |  |  |  |
| ክፍል 10. የቤተሰቡ የምግብ ዐመጋገብ | |  |  |  |
| **Now I would like to ask you about the types of foods that you or anyone else in your household ate yesterday during the day and at night either separately or combined with other foods.** | | | | |
| ቤተሰቡ በ 24 ሰዐት ዉስጥ የተመገበውን የምግብ ዐይነት የሚገለፅ መጠይቅ (ቤተሰቡ ቁርስ፣ምሳ፣ዕራት የሰራውን ወይም የተመገበውን ምግብ ጠይቅ የሚስማማውን ቦታ ዐመልክት) | | | | |
| 1000 | **Were there any foods that were not prepared for the in the house**  **because it was a fasting day?** |  |  |  |
|  | Qatume ikkewo daafira loonsoyikki sagale no | 0. [_]No | 0. [_]Dee’ni | 0. [_]አይደለም |
|  | ጾም ስለሆነ ያልተሰራ ምግብ ዐለ | 1. [_]Yes | 1. [_]Eewa | 1. [_]አዎን |
| 1001 | **Could you tell me the types of foods that were prepared in the house and that you or anyone else in your household ate?** |  |  |  |
|  | Loonsoyi sagale kulatoe | [_]Breakfast |  | [_]ቁርስ |
|  | ለቤተሰቡ የተሰራውን የምግብ ጊዜ ትነግሪኛለሽ | [_]Lunch |  | [_]ምሳ |
|  |  | [_]Dinner |  | [_]ዕራት |
|  |  | [_]Others |  | [_]ሌላካለ ጥቀስ/ሽ |
| 1002 | **Any bread, rice, pasta, biscuits, or any other foods made from millet, sorghum, maize, rice, wheat?** |  |  |  |
|  | Daabbo,paarta,ruuze,koshoro raino sagale woy ajjunni ,badalatenni ,hayixunni,qamadetenni,bashanqunniy loonsoonni sagale woy wolu quminni,xawisi | 0. [_]No | 0. [_]Dee’ni | 0. [_]አይደለም |
|  | ዳቦ፤ ፓስታ፤ ሩዝ፤ ብሰኩት፤ ኩኪሰ፤ ወይም ማንኛውም ነገር ከአጃ ከበቆሎ ገብሰ፤ ሰንዴ፤ ማሸለ፤ወይም ሌላ አህል ዘር የተሰራ | 1. [_]Yes | 1. [_]Eewa | 1. [_]አዎን |
| 1003 | **Any potatoes, bulla, kocho or any other food made from roots or tubers?** |  |  |  |
|  | Maxaaxeesla, diinicha, boyina,lawinore saga’lino? | 0. [_]No | 0. [_]Dee’ni | 0. [_]አይደለም |
|  | ማንኛውም ነጭ ድንች፤ ቦይና፤ እንሰት (ማንኛውም ነጭ ስራስር) | 1. [_]Yes | 1. [_]Eewa | 1. [_]አዎን |
| 1004 | **Any vegetables?** |  |  |  |
|  | Wole aye ataakilte saga’lino? | 0. [_]No | 0. [_]Dee’ni | 0. [_]አይደለም |
|  | ማንኛውም አታክልት | 1. [_]Yes | 1. [_]Eewa | 1. [_]አዎን |
| 1005 | **Any fruits?** |  |  |  |
|  | Wole aye gumma | 0. [_]No | 0. [_]Dee’ni | 0. [_]አይደለም |
|  | ማንኛውም ፍራፍሬ | 1. [_]Yes | 1. [_]Eewa | 1. [_]አዎን |
| 1006 | **Any beef, pork, lamb, goat, rabbit wild game, chicken, duck, or other birds, liver, kidney, heart, or other organ meats?** |  |  |  |
|  | Bootu,mancheemete,gereewo,mellenna hilleessa,Lukko,daakiyye, Afale,mule,wodana,woy wole godowu giddo malla? | 0. [_]No | 0. [_]Dee’ni | 0. [_]አይደለም |
|  | የበሬ፤ የበግ/የፍየል የአሳማ,የጥንቸል ,ዶሮ፤ እርግብ/ወፍ (ጉበት፤ ኩላሊት፤ የልብ ወይም ማንኛውም የውሰጥ ሰውነት ክፍል) | 1. [_]Yes | 1. [_]Eewa | 1. [_]አዎን |
| 1007 | **Any eggs?** |  |  |  |
|  | Ayee quuphe | 0. [_]No | 0. [_]Dee’ni | 0. [_]አይደለም |
|  | ማንኛውም እንቁላል | 1. [_]Yes | 1. [_]Eewa | 1. [_]አዎን |
| 1008 | **Any fresh or dried fish or shellfish?** |  |  |  |
|  | Ayee qilxi’me | 0. [_]No | 0. [_]Dee’ni | 0. [_]አይደለም |
|  | ማንኛውም አሳና የአሳምርት | 1. [_]Yes | 1. [_]Eewa | 1. [_]አዎን |
| 1009 | **Any foods made from beans, peas, lentils, or nuts?** |  |  |  |
|  | Aye segale baqeluuni, atarunni, qibaatete qumma shumburunni qixxeessinoonni sagale saga’lino? | 0. [_]No | 0. [_]Dee’ni | 0. [_]አይደለም |
|  | ማንኛውም ምግብ ከ ባቂላ፤አተር፤ ምሰር ወይም ሌላ ጥራጥሬ | 1. [_]Yes | 1. [_]Eewa | 1. [_]አዎን |
| 1010 | **Any cheese, yogurt, milk or other milk products?** |  |  |  |
|  | Ayibe/geinto saga’lino? | 0. [_]No | 0. [_]Dee’ni | 0. [_]አይደለም |
|  | ማንኛውም አይብ፣እርጎ፣ወተት ወይም ሌላ የወተት ምርቶች | 1. [_]Yes | 1. [_]Eewa | 1. [_]አዎን |
| 1011 | **Any foods made with oil, fat, or butter?** |  |  |  |
|  | Zayitetenni woy buurumi loonsoonni sagala | 0. [_]No | 0. [_]Dee’ni | 0. [_]አይደለም |
|  | በዘይት ወይም በቅቤ የተሰራ ምግብ | 1. [_]Yes | 1. [_]Eewa | 1. [_]አዎን |
| 1012 | **Any sugar or honey?** | 0. [_]No | 0. [_]Dee’ni | 0. [_]አይደለም |
|  | ማንኛውም ስኩዋርና ማር | 1. [_]Yes | 1. [_]Eewa | 1. [_]አዎን |
| 1013 | **Any other foods, such as condiments, coffee, tea?** | 0. [_]No | 0. [_]Dee’ni | 0. [_]አይደለም |
|  | ቡናና ሻይ | 1. [_]Yes | 1. [_]Eewa | 1. [_]አዎን |
| 1014 | **Alchol drink** |  |  |  |
|  | Diribisanno ago (Birra, Xesiixella,Areqe | 0. [_]No | 0. [_]Dee’ni | 0. [_]አይደለም |
|  | ዐልኮል መጠጦች (ቢራ፣ ዐረቄ፣ጠጅ፣ጠላ) | 1. [_]Yes | 1. [_]Eewa | 1. [_]አዎን |
| 1015 | **Others specify** |  | | |
|  | Welere |  |  |  |
|  | ሌላ ካለ ዘርዝር |  |  |  |
|  |  |  |  |  |
