# Supplementary material for: Efficacy of processed amaranth-containing bread compared to maize bread on hemoglobin, anemia and iron deficiency anemia prevalence among two-to-five year-old anemic children in Southern Ethiopia: A cluster randomized controlled trial
Source: PLoS One. 2020 Sep 28;15(9):e0239192. doi: 10.1371/journal.pone.0239192 (PMC7521750; doi:10.1371/journal.pone.0239192)
Supplement: S2 Appendix — (DOCX) [file pone.0239192.s004.docx]

**Preventing iron deficiency anemia: Evaluation of amaranth grain supplementation to 24.0-59.9 month old children in southern Ethiopia, a randomized controlled trial**


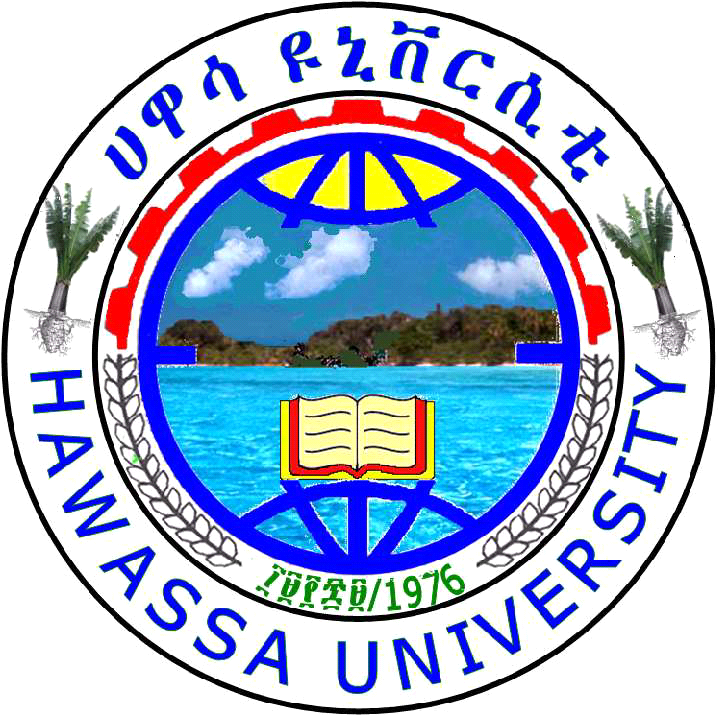

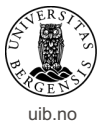


**PhD Protocol**

**By: Alemselam Zebdewos (MSc)**

**Main supervisor: Prof.Ingunn Marie S. Engebretsen**

**Co-supervisors: Prof.Bernt Lindtjorn and Dr. Eskindir Loha**

**Hawassa University School of Medicine and Public Health/ University of Bergen, Centre for International health**

**Hawassa, Ethiopia**

**June, 2016**

**Acknowledgement**

First of all I would like to express my sincere thanks and appreciation to South Ethiopia Network of Universities in Public Health (SENUPH) member universities and University of Bergen for their role in effecting network among the universities which laying the ground for this research.I would like to extend my deepest thanks to Prof. Bernt Lindtjørn and Dr. Eskinder Loha for initiating and founding such magnificent link between universities.

I am deeply indebted to my major Supervisor, Prof. Ingunn Marie S. Engebretsen for her unreserved passionate assistance and guidance I went to take this opportunity to appreciate her passion and energy to assist. I would like to give a special thanks to my Co-supervisor to Prof. Bernt Lindtjørn to his friendly advice, encouragement and support from the beginning of protocol writing. Next I would like to extend my thanks to my Ethiopian Co-Supervisor Dr.Eskinder Loha for his effort to comment the protocol. At the end I went to extend my thank to my whole family specially to my lovely husband Damte Data for his encouragement, advice and support without his support I wouldn’t reach in this level thank you Da for your help I love you!

# Summary

**Introduction:** Malnutrition is a public health problem in Ethiopia. Malnutrition can affect children’s cognitive, behavioral and motor development. Further, healthy development and educational achievement can be compromised due to micronutrient deficiencies including iron deficiency. Different studies show that amaranth grain has better levels of macro and micronutrients especially iron to address the widespread malnutrition problem than other frequently used crops. This useful plant is widely grown in the research area, but it considered as a weed rather than a food crop by the community. Furthermore, adequate research has not been conducted in Ethiopia on amaranth’s potential to contribute in reducing the malnutrition problem. This research is initiated to look at amaranth and its potential in reducing iron deficiency anemia among children. The research will inform the public at large, policy makers and the academicians as it deals with an underutilized nutritious crop that has the potential to contribute to reduce the malnutrition problem in the country.

**Objective:** To assess the iron deficiency status of children and to evaluate the effect of an amaranth containing bread consumption on the anthropometry and iron deficiency status of children in the age of 24.0-59.9 months.

**Methodology:** A cross sectional study and a randomized controlled trial will be done. For the survey, random sampling will be used to select 340 children. Hemoglobin, serum ferritin, C- reactive protein and anthropometric measurements will be taken and used as the baseline data for the experimental study. In the randomized controlled trial, 100 children with hemoglobin level of less than 11 mg /dl and above 7 mg/dl (mild and moderate anemia) will be recruited into the trial after informed consent. Severely ill individuals will be referred to Hawassa Referal Hospital. Kebele-stratified simple random sampling will ensure 1:1 allocation to each group. Sequentially numbered, opaque, sealed envelopes will be used to assure concealment of allocation. The study subjects and parents (other caregivers in the absence of parents) will be kept uniformed about the trial allocation. Statistical analysis will be done using the latest SPSS version and ENA for SMART software. The survey will present descriptive statistics including frequencies, means, ranges and demographic characteristics of the population. The trial will be analyzed according to the intention-to-treat principles using logistic crude and site adjusted regression models with a mean difference in Hemoglobin as the primary outcome measure.

**Conclusion:** This research project has the potential to be hypothesis generating and will inform policy makers and scientists about amaranth’s potential to combat malnutrition specifically iron deficiency in the area.

**Keyword: Amaranth, anthropometry, iron deficiency anemia**

Table of Contents

[Summary iii](#_Toc471130462)

[Acronyms viii](#_Toc471130463)

[1. Introduction 1](#_Toc471130464)

[1.1. Background 1](#_Toc471130465)

[1.2. Statement of the problem 3](#_Toc471130466)

[1.3. Study justification 4](#_Toc471130467)

[2. Aim 5](#_Toc471130468)

[2.1. Research questions: 5](#_Toc471130469)

[2.3. Hypothesis of this study 5](#_Toc471130470)

[3. PICO 6](#_Toc471130471)

[3.1. General objective one 6](#_Toc471130472)

[4.2. General objective two 7](#_Toc471130473)

[5. Method 8](#_Toc471130474)

[4.1. Study Area 8](#_Toc471130475)

[5.2. Study population 8](#_Toc471130476)

[5.3. Study period 9](#_Toc471130477)

[5.4. Study subject 9](#_Toc471130478)

[5.5. Sample Size 9](#_Toc471130479)

[5.6. Participants 10](#_Toc471130480)

[5.7. Inclusion criteria 10](#_Toc471130481)

[5.8. Exclusion criteria 10](#_Toc471130482)

[5.9. Sampling Technique 11](#_Toc471130483)

[5.10. The intervention 11](#_Toc471130484)

[5.11. Recipe, baking, packing and distribution 12](#_Toc471130485)

[4.13. Study variables 14](#_Toc471130486)

[4.14. Statistical methods 14](#_Toc471130487)

[4.15. Pre-testing of data collection tools 15](#_Toc471130488)

[5. Purpose and potential of the study 16](#_Toc471130489)

[6. Ethical consideration 17](#_Toc471130490)

[6.1. Individual advantage and disadvantage from the research 17](#_Toc471130491)

[6.2. Advantage and disadvantage of the research for the community and society 18](#_Toc471130492)

[7. Budget break down 21](#_Toc471130493)

[8. References: x](#_Toc471130494)

**Figures**

[Figure 1 Amaranth Picture collected from the study area 12](#_Toc459903348)

[Figure 2 Steps of homemade amaranth processing 23](#_Toc459903349)

[Figure 3 Proposed trial profiles 35](#_Toc459903350)

Tables

[Table 1 Nutrient Content of Formulated Bread Compare to Maize Bread 23](#_Toc459903351)

[Table 3Trial Staff distribution based on blindness 26](#_Toc459903352)

[Table 4 Trial Schedule 32](#_Toc459903353)

# Acronyms

| BC | Beta-Carotene |
| --- | --- |
| CRP | C – Reactive Protein |
| EPHI | Ethiopian Public Health Institute |
| FE MNP | Ferro Magnetic Nano particles |
| FGD | Focus Group Discussion |
| FVS | Food Varity Scores |
| HDD | House Hold Dietary Diversity |
| HAZ | Height For Age Z-Score |
| IDA | Iron Deficiency Anemia |
| IDD | Individual Dietary Diversity |
| IYCF | Infant Yange Child Feeding |
| MND | Micro Nutrient Deficiency |
| MUAC | Mid Upper Arm Circumference |
| PLWHA | People Living With HIV/AIDS |
| RDA | Recommended Dietary Intake |
| RES | Reticule Endothelial System |
| SRL | Sustainable Rural Livelihoods |
| WHO | World Health Organization |

# 1. Introduction

## 1.1. Background

Ethiopia is one of the developing countries in the world where children and their mothers suffer most from poor health and nutrition. Malnutrition is the leading cause of death among children aged under 5 years in Ethiopia ([1](#_ENREF_1)). The country is challenged with the highest rates of stunting and underweight in the world. The recent Ethiopian Demographic Health Survey ([2](#_ENREF_2)) reported that almost half (44%) of Ethiopian children under five years of age were stunted (an indication of chronic malnutrition) and close to one in four (21%) were severely stunted. 10% of children under five years are wasted (an indication of acute malnutrition) which is considered a high prevalence according to WHO’s classification system ([3](#_ENREF_3)).

Micronutrient deficiency is a major contributor to childhood morbidity and mortality in Ethiopia. Iron, Iodine, vitamin A and zinc are recognized as ‘‘problem nutrients’’. Anemia is one of the major public health problems in Ethiopia. According to available information it is indicated that about 44% of children under five years are anemic in Ethiopia ([4](#_ENREF_4), [5](#_ENREF_5)). The World Health Organization (WHO) considers anemia prevalence over 40 percent in a population to be a major public health problem. Low iron intake in children is associated with increased morbidity and mortality as well as limited physical and cognitive development. IDA can result in compromised cognitive, behavioral and motor development as well as sub-optimal educational achievement ([6](#_ENREF_6), [7](#_ENREF_7)). Inadequate iron intake is responsible for more than half of anemia cases as diets consisting of mostly staple food dominated by starchy cereals are common for most of the poor people in Ethiopia. The diets are typically low in lipids, protein, vitamins and minerals ([2](#_ENREF_2), [6](#_ENREF_6)).

Iron is one of essential micronutrient required largely for children aged 2 to 5 years of age. An intake of 7-10 mg of iron is the recommended daily intake, children should consume via iron-rich foods. The available data in Ethiopia indicates that the majority of children are not consuming iron-rich foods. Only 5 percent of children consume meat, fish or poultry, which are bio-available, iron rich foods ([2](#_ENREF_2)). A study among school-aged children from west Ethiopia found an anemia prevalence of out 43.7% of which 37.4% was Iron Deficiency Anemia (IDA) which was associated with not-consuming protein source foods, dairy products, and discretionary calories ([5](#_ENREF_5)). The other predictors of IDA were not-consuming dairy products, not-consuming discretionary calories, low family income and parasitic infections ([5](#_ENREF_5)).

There are different methods to solve the problem of iron deficiency such as food fortification and supplementation. Iron supplementation of pregnant women, which address a limited number of women is the only IDA intervention currently implemented in Ethiopia including the research area to combat iron deficiency anemia. An increasing number of studies reported that iron supplementation intervention increases morbidity and causes unfavorable shifts in the gut microbial composition along with an increase in intestinal inflammation, particularly in children with a high infectious disease burden ([8](#_ENREF_8)). Additionally, food fortification and supplementation is expensive and cannot be afforded by the majority of the poor Ethiopian population. Supplementation has so far not shown to be a sustainable solution to prevent iron deficiency at group level. Amaranth contains protein and micronutrient of unusually higher amount than staple cereals ([9](#_ENREF_9)). Amaranth grain contains twice the level of calcium than in milk, five times the level of iron in wheat, higher potassium, phosphorous and vitamins A, E and folic acid than cereal grains. It can be cultivated for both its seeds, which are used as a grain and its leaves which are used as vegetables ([9](#_ENREF_9), [10](#_ENREF_10)).

Even though amaranth contains quality amino-acids, high iron and other micro-nutrients, it also has a high concentration of phytic acid that can reduce the bio-availability of iron. A recent study from Kenya ([10](#_ENREF_10)) indicated that amaranth has no effect on the iron status of children due to high phytate to iron ratio. However, research conducted in Ethiopia and Kenya showed that homemade processing such as soaking and germination of the grain amaranth could reduce phytate to an acceptable phytate to iron ratio level that can contribute to increased bio-availability of iron ([11](#_ENREF_11), [12](#_ENREF_12)).

Amaranth is producing cereal like gluten-free seeds, which has an outstanding agronomic performance owing to its resistance to drought, hot climate and pests. It can grow in all-weather conditions specially the place where maize can grow. It also gives higher yields than maize. It doesn’t require high nitrogen like maize but responds well to fertilization. Amaranth produce a large amount of biomass in a short period of time and therefore has the potential to contribute a substantial increase in world food production grain yields ([13](#_ENREF_13)).

## Statement of the problem

Anemia is a global health problem. Iron deficiency is the most common micronutrient deficiency (MND) worldwide and most common in developing countries which lead to microcytic anemia. Anemia is a major public health problem in Ethiopia and it is more common among groups with low socio economic status ([6](#_ENREF_6), [14](#_ENREF_14)).The major cause for IDA is insufficient dietary iron intake, compounded by high incidence of malaria and other parasitic diseases. Ethiopian diet is mainly composed of cereals (maize, sorghum), tubers and root crops (ensete, potatoes, and sweet potatoes) which are low in iron and other micronutrients([15](#_ENREF_15)). Despite a large livestock population, the food supply of animal products such as meat is very limited and consumption of these products is especially low in rural areas because it is expensive. Thus, there is a need to find other sustainable contributors to the diet that can improve the nutritional status of children. Therefore, this study aims to evaluate the effect of processed amaranth grain recipes consumption to reduce iron deficiency anemia and improve anthropometric status among children in southern Ethiopia.

## Study justification

The grain amaranth has the better level of nutrients and it could be a better choice to use for food formulation ([16](#_ENREF_16), [17](#_ENREF_17)) at the household level and at factories than maize. Amaranth is draught resistant, can be grown in almost all parts of the country and has the ability to give more yield than maize and other staple crops in the area. So amaranth is one alternative to address the widespread protein and micronutrient deficiencies with low cost ([18](#_ENREF_18)).

Even though this prospective crop is widely grown in Ethiopia, it is not used as a food and food supplement; rather it is considered a useless weed. Therefore, this research will create new insight in amaranth’s ability to reduce malnutrition, specifically iron deficiency anemia. It also tests out a low cost iron rich recipe relevant for the Ethiopian society that cannot afford fortified foods or animal products.

# Aim

To reduce iron deficiency anemia in children 24.0 up to 59.9 months of age by using amaranth grain in southern Ethiopia.

## **2.1. Research questions**:

1. What is the prevalence of iron deficiency anemia defined as Hemoglobin below 11mg/dl and serum ferritin below 12µg/L among children from 24.0 to 59.9 months of age in southern Ethiopia?
2. Does consumption of 150 mg bread daily containing 70 mass percentage amaranth grain increases mean hemoglobin level by 0.7mg/dl among children 24.0-59.9 months of age compared to equivalent amount of 100 percent maize bread in southern Ethiopia?
3. Is Amaranth grain culturally acceptable and how is the policy direction towards amaranth grain to use as staple food in Ethiopia?

## 2.2. Hypothesis of this study

**HO:** Feeding 150gm of bread containing 70 mass percentage amaranth grain and 30 mass percentage chickpea per day for mild and moderate anemic children 24.0 up to 59.9 months of age for 6 months will not increase the mean hemoglobin level by 0.7g/dl compared to 150gm of 100 percent Maize containing bread in Tulla sub city southern Ethiopia.

**HA**: Feeding 150gm of bread containing 70 mass percentage amaranth grain and 30 mass percentage chickpea per day for mild and moderate anemic children 24.0 up to 59.9 months of age for 6 month will increase the mean hemoglobin level by 0.7g/dl compared to 150gm of 100 percent maize bread in Tula sub city ,southern Ethiopia.

# PICO

P- Children aged 24.0 -59.9 months in southern Ethiopia, Tulla sub city, with mild and moderate anemia

I - Daily feeding of 150g bread containing 70 mass % amaranth grain and 30 mass % mashed chickpea

C -Daily feeding of 150g bread containing 100% maize

O- Change in hemoglobin level, change in ferritin status, changes in anthropometric status.

The research questions will be answered in three subsequent papers and the general and specific objectives are given below for the three papers.

## General objective one

To estimate the prevalence of iron deficiency anemia in children age 24.0-59.9 month in Tulla sub city, South Ethiopia.

**Specific objective**

1. To describe the distribution of hemoglobin levels among children 24.0-59.9 months of age in Tulla sub-city South Ethiopia including mean, median, ranges and 95 % confidence intervals (95% CI) and percentage of children falling under the category of mild and moderate anemia.
2. To describe the distribution of ferittin levels of children 24.0-59.9 months of age in Tulla sub city south Ethiopia including mean, median, ranges and 95 % confidence intervals (95% CI) and determine the percentage of children who are mild- and moderate anemic with low ferritin levels.
3. To describe the anthropometric indices of children 24-59 month of age in Tulla sub city south Ethiopia
4. To assess amaranth acceptability and policy direction in Tulla sub city south Ethiopia.

## General objective two

To evaluate if feeding children with either 150gm bread containing 70mass% amaranth grain and 30mass% chickpea per day for 6 month will increase the hemoglobin level of mild and moderate anemic children 24.0 up to59.9 months of age by 0.7g/dl compared with children in equivalent amount of 100% maize bread in Tulla sub city south Ethiopia .

**Specific objective**

1. To compare the hemoglobin values in children receiving amaranth grain containing bread and children receiving maize bread in Tulla sub city South Ethiopia.
2. To compare the ferritin values in children receiving amaranth grain containing bread with children receiving maize bread in Tulla sub city south Ethiopia.
3. To compare the anthropometry indices in children receiving amaranth grain containing bread with children receiving maize bread in Tulla sub city south Ethiopia.
4. To compare incidence of childhood illness in children receiving amaranth grain containing bread with children maize bread in Tulla sub city south Ethiopia.
5. To compare cost effectiveness of amaranth containing bread on the prevention of iron deficiency anemia in Tulla sub city south Ethiopia
6. To compare compliance and adherence of mother between amaranth containing bread and maize bread groups.

# Method

## 4.1. Study Area

Ethiopia is a large, country in Eastern Africa with a population of over 90 millions. More than 80% of its population live in the countryside and make their living through traditional agricultural practices. The proposed research will be conducted in the southern part of Ethiopia in Hawassa city administration, Tulla sub city. The city is located 270 km from Addis Abeba, the capital city of Ethiopia. Currently Hawassa city consists of 8 sub cities and 32 villages/Kebelles each having its own administration offices.Tulla sub citiy is on e of the sub city of Hawassa city administration. Most part of the Tulla sub city is rural, the current projected total population of Hawassa city is 328 thousand, of this 169 thousand are males and 159 thousand are females. The estimated numbers of the households are 70 thousand. 51 thousand are children under five years. 46 thousand are aged 24-59 months. Annual average temperature is 12-27^0^C and the annual average rain fall is 500-1800 mm. The main crops produced in the area that maize, hallicot beans and Irish potatos. Amaranth is growing as a wild plant in most areas of Tulla sub city and the leave part is used by the community as a vegetable.

## Study population

The source population of this study will be children aged 24-59 months of age residing in Tulla sub-city of Hawassa city administration. Tulla sub city is divided in to 8 kebelles. From this kebelle Cheffe cote jebesa and Gara riketa will be purposively select for the project site because they are near to the city, has transport accesses and amaranth grows widely in the area as wild plant.

## Study period

The proposed study period will be from month 12 2016 to month 3 2017.

- 1. Study subject 24.0 months – 59.9 months of age child who live in Tulla sub city southern Ethiopia.

## Sample Size

The base line data (survey part) will be used as a basis for the trial part. After completing the survey part the trial sample will be recruited from the survey study participants, in this way the survey will be providing the baseline data for the trial. The survey sample size will be 340 considering a need for 100 trial participants, 33% of mild and moderate anemia prevalence in south Ethiopia, 0.5 degree of precision and 95% confidence intervals. The survey sample size may be increased till the numbers of eligible individuals are identified for the trial if anemia is less prevalent than 33%.

The sample size of the trial is calculated by using Stata I.C. 14 software using the sample size command, considering 0.7 mean difference,1 standard deviation, power 90% and attrition rate 12% the total sample size will be 99 (100) in the two groups. The total sample will be divided in to the amarnth70%/chickpea30% versus the maize 100% groups. Thus it will be 50 in the amaranth/chickpea group and 50 in the maize group ([2](#_ENREF_2)).

## Participants

The target population of this study is children 24.0-59.9 months of age with mild and moderate anemia in Tulla sub-city, southern Ethiopia. Below is described the inclusion and exclusion criteria of the trial participants.

## Inclusion criteria

- Children 24 months up to 59 months
- Children with hemoglobin level 7 to <11 mg/dl
- Currently residing in the study area, Tulla sub-city of Hawassa city administration, having lived in the study area for at least 6 month prior to the study and who are planning to continue to be there for the next 1 year.
- Guardians provide informed consent and the children ascent.

## Exclusion criteria

- Children with chronic illness like HIVAIDS, tuberculosis, cancer and any other unspecified chronic illness
- Severely malnourished children < -3 Z score
- Children who are taking iron supplements
- Children who got a blood transfusion in the last 6 months
- Children who are unable to collaborate (any somatic or mental disability making collaboration difficult more than expected for the age range)
- Children with repeated malaria who are diagnosed as positive for malaria at least 3 times the last 3 months
- Any suspected hematological disease

## Sampling Technique

All children age 24.0-54.0 months will be registered in the selected Kebelles. From the registered children 340 children will be select by using simple random selection for survey part.

After assessing Hgb, 100 children with mild and moderate anemia status will be included in the trial. The included children will be allocated randomly 1:1 to amaranth70% chickpea30% versus maize100% 150g daily bread. Single randomization stratified by Kebelle will be used.

Randomization will be done using random org sequence free soft ware. Researchers not involved in the field work in Hawassa University will provide the sequence; researchers at the partner institution UiB will provide the seed number.

## The intervention

Eligible participants will be assigned randomly in to amaranth70% chilckpea30% versus maize100% 150mg daily bread groups. After randomization they will be given a card where it is stated their id-number and study participation. After assigning the participants to one of the two groups the respective 150 gm of the correct bread will be provided every day for 6 month in each group. The participants will be assigned to a ‘distributor’ who will be kept uninformed about the content of the bread he or she is distributing.

The trial will be double blinded: it means that the one receiving the amaranth70%/chilckpea30% or maize100% bread, the one distributing it and the data collectors will be kept uninformed about the trial allocation. To make the color of both bread types similar, maize will be processed (roasted) to change the color (darker). Bread distributors will be different for amaranth70% chickpes30%group and the maize100% group. Both bread distributors will get the bread from coordinator and they will distribute separately according to the trial allocation.

## Recipe, baking, packing and distribution

The recipe will be prepared based on the recommended dietary intake (RDA); according to RDA 150 gm of bread (70% amaranth with 30% chickpea) will provide approximately 50% of recommended amount of iron per day. The 70% of amaranth and 30 % chick pea bread will provide 15.16 mg of iron per 100 gm bread ([11](#_ENREF_11)). So the bread will meet the daily iron requirement for amaranth70%/chickpea30% group by estimating the absorption of iron at 15% -20% ([19](#_ENREF_19)). Both maize and amaranth grain flour will be prepared and packed separately. After packing each package will be labeled with a pre-printed sticker containing the id number. Two boxes per kebelle will be packet and labeled with the respective bread distributor name. In this way we ensure that the distributor gets the right box of bread and the participant the right bread. It will further be easy to keep track of ‘unopened’/’returned’ bread packages at the coordinator level every day in case of refusals or absenteeism.

Amaranth will be soaked in water by adding 5 ml of lemon juice per 100ml of water for 24 hours and germinate for 72 hours. After sun drying it will be roasted and milled with a local electrical mill ([11](#_ENREF_11)).This will decrease the phytate level and is expected to increase iron absorption in the gut. For the maize100% group the same size of bread which is prepared from roasted maize will be provided.

Both amaranth and maize bread will be made in separate local commercial bread making factories to decrease contamination or mixing of the bread. The bread will contain 5ml oil and 5ml sugar. The target daily intake of bread will be 150g for all children, considered to be an amount that they could comfortably consume in one session. The micronutrient and energy level is specified in Table 1below.

Table 1 Nutrient Content of Formulated Bread Compare to Maize Bread

| 100g | Protein mg/100gm | Fat mg/100gm | Iron mg/100gm | Calcium  mg/100gm | Zinc mg/100g | Energy  K.cal |
| --- | --- | --- | --- | --- | --- | --- |
| 100%maize | 7.58 | 4.44 | 4.31 | 24.82 | 2.47 | 386 |
| 70%amaranth and 30% c.pea | 16.39 | 7.08 | 15.16 | 244.71 | 2.59 | 404 |


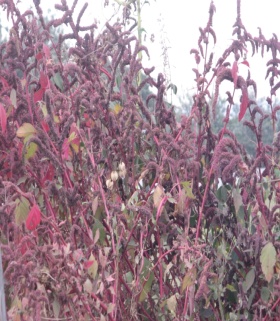

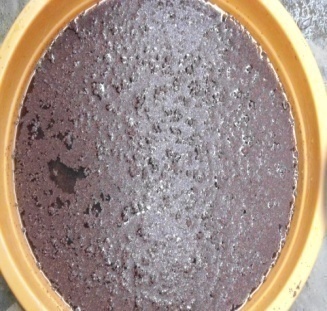

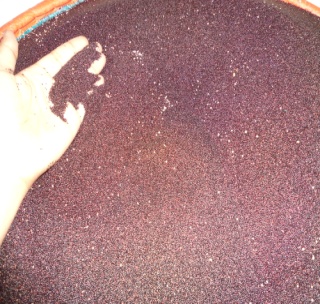

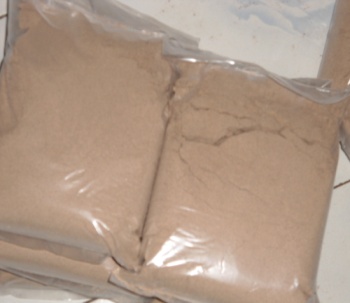


Flour

Roasted

Socked

Amaranth ingarden

Figure 2 Steps of homemade amaranth processing

# Study variables

The primary outcome measure of this study will is hemoglobin of children and serum ferritin. The secondary outcomes are anthropometry measures (height/length, weight and mid-upper-arm circumference) (anthropometric z- scores will be derived based on WHO growth standards 2006 (give reference)), conjunctiva pallor and child morbidity pattern including hospitalization, diarrhea, cough and fever (type, frequency, duration and symptoms of illnesses). Potential effect modifiers and confounders will be captured to describe the population and look at the successfulness of randomization (sex, education level, occupation and income), dietary practices (number of meals and frequency of food consumption) including use of amaranth in the diet and type of staple foods.

# Statistical methods

Data will be entered in to epidata.dk and transferred in to SPSS (version 20.0) software for analysis. ENA for SMART software will be used to convert anthropometric data (weight, height) to z-scores and percent of the median in terms of wasting, underweight and stunting. Data on hemoglobin and serum ferritin levels will be described by using descriptive statistics (mean and percentages) and will be summarized by using tables and figures.

Data on dietary practices, nutritional status, morbidity patterns and serum levels will be described by using descriptive statistics (mean and percentages) and will be summarized by using tables and figures. The descriptive variables will be presented by arm to assess successfulness of the randomization and to describe the study population. Intention to treat analysis using logistic analysis for the main trial outcome hemoglobin will be done and adjustment will only be done for skewed and potentially confounding factors.

Further, descriptive statistics including morbidity prevalence and dietary practices and food diversity will be described from the survey. The relationships between variables such as sex and type of illness which are categorical in nature will be established by using chi-square. Paired t - test will be used to determine if there is a significant difference between the dietary practices (number of meals, diet diversity score), nutritional status, morbidity patterns (frequency and duration of illness), mean hemoglobin level and serum ferritin levels. Simple regression analysis will be used to determine the contribution of dietary practices and morbidity patterns to nutritional status hemoglobin and serum ferretin levels. Multiple regression analysis will be used to determine the extent to which dietary practices and morbidity patterns predict hemoglobin serum ferritin and nutritional status.

# Pre-testing of data collection tools

The pre-testing samples will include10 caregivers of children under five. The sample will be from the study area but not targeted for the main study. After pre-testing, questions will be edited based on information and the tools will be adjusted accordingly.

Table 4 Trial Schedule

|  | Month 1 | Month 2 | Month 3 | Month4 | Month 5 | Month 6 | Month 7 | Month 8 |
| --- | --- | --- | --- | --- | --- | --- | --- | --- |
| **Preparation** |  |  |  |  |  |  |  |  |
| Grain collection | X |  |  |  |  |  |  |  |
| Flour preparation | X |  |  |  |  |  |  |  |
| Data collectors training | X |  |  |  |  |  |  |  |
| Pre testing | X |  |  |  |  |  |  |  |
| Census (children registration) |  | X |  |  |  |  |  |  |
| Informed consent |  | X |  |  |  |  |  |  |
| **Survey** |  | X |  |  |  |  |  |  |
| Socio demographic data |  | X |  |  |  |  |  |  |
| Haemoglobin level |  | X |  | X | X |  | X | X |
| CRP |  | X |  |  | X |  |  | X |
| Serum ferritin |  | X |  |  |  |  |  | X |
| Anthropometry measure |  | X |  |  | X |  |  | X |
| Clinical examination |  | X |  |  | X |  |  | X |
| Illness history |  | X |  |  | X |  |  | X |
| **Trial** |  |  |  |  |  |  |  |  |
| Eligibility of child |  |  | X |  |  |  |  |  |
| Randomization |  |  | X |  |  |  |  |  |
| Deworming |  |  | X |  | X |  |  |  |
| Feeding with bread |  |  | Every day | | | | | |
| **Follow up visit** |  |  |  |  |  |  |  |  |
| Clinical assessment |  |  | **Monthly** | | | | | |
| Illness history |  |  |  |  |  |  |  |  |
| Food frequency data |  |  |  |  |  |  |  |  |
| Compliance and adherence |  |  |  |  |  |  |  | X |

# Purpose and potential of the study

The purpose of the study is to contribute a valuable input for the next or upcoming five year growth and transformation strategic plan of the government on the prevention of malnutrition strategy in Ethiopia. Since half of the causes of child death are related with malnutrition, this study will show further direction to prevent malnutrition in Ethiopia by producing information for policy makers. The information from this study will provide a potential direction to formulate child formula in the factory and farmers in producing amaranth crop. So this research could create a good opportunity to promote the amaranth grain among the community. This will be one step to combat malnutrition and hunger in the area. It can also show us further needs to study the use of amaranth.

# Ethical consideration

Since Amaranth is edible and has no toxicity effect ([16-18](#_ENREF_16), [20](#_ENREF_20), [21](#_ENREF_21)) ethical clearance will be obtained from the Institution Ethical board of Hawassa University and Regional ethical community of Western Norway. The required data will be collected after getting official permission from Tulla sub city Health bureau. Informed written consent will be obtained from the study participants. Each respondent will be informed about the objective of the study. All record or information will be kept locked to use when it is needed. The person identification information will be kept separate from the data file for analysis and only completely anonymous data files will be uploaded for co-authors, reviewers and in a public repository.

## Individual advantage and disadvantage from the research

This trial is expected to contain no risk from the intervention. The 70%amaranth/30%chickpea bread group is expected to benefit to get a better nutritional status at the endpoint at the study and the 100% maize bread group is expected to benefit from a comparable caloric intervention but of lower micronutrient value. Even though there is strategic plan to reduce anemia at 2015 almost by half but there is no active program in the area to identify and supplement moderate and mild anemia cases. Moderate and mild anemia is asymptomatic they may not be diagnosed without laboratory. If the children are not participate in the study they may not get chance to be diagnosis as anemic, so to be diagnosis is one of the benefit which will create a chance to be treated at the end of the study or before the beginning of the study by refusing participation in the study. Sever anemia and seriously ill individual identified they will be referred to hospital.

At the end of the study all children who participated in the study will receive iron supplementation. Every month physical examination will be done to control sever progression, if individual progressed to sever anemia they will be referred to hospital. At the mid of the study hemoglobin and ferretine level will be tested to control any harm that the study may cause on the child. Health education will be given every month during follow up this may help them how to keep their health and feeding style. Blood sample collection is an invasive procedure and it may cause discomfort and pain for children. The 5ml sampling three times is the minimum data collection we can do in order to get meaningful data. Even though it is invasive procedure the study will use standard precaution to prevent infection transmission by using an experienced professional laboratory technician.

## Advantage and disadvantage of the research for the community and society

There is no foreseen disadvantage to the community and society except increased driving, however, this latter point is believed to be relatively minimal. This research will benefit the community by providing information about potential advantages of amaranth. This information may stimulate the farmer to produce amaranth and which may help them to improve their household food needs and to generate income. At societal level the dissemination of the result will create awareness for policy makers to look at the hidden resource to fight hunger. Since amaranth is drought resistant and giving high yield compared to local staple crops it may be one of the solutions to fight hunger by promoting amaranth cultivation in Ethiopia. In the scientific society it will create new information concerning the unresolved question about the effect of amaranth on the prevention of iron deficiency. This research will look at processed amaranth to answer this uncertainty. To conclude, comparing the risks and benefits in this study the benefits is larger than the risks.

The research area will be select purposively

Simple random sampling to recruit 340childrenfrom population

340 children will be identified Using Simple random sampling

The research area will be select purposively

Hemoglobin, serum ferritin, anthropometry, socio demographic data will be assessed

100 Children with low hemoglobin level < 11 And > 7 mg/dl

50 children exposure group

50 children control group

Amaranth containing bread 150mg per day for 6 month

Processed maize 150mg/day bread per day for 6 month

Mean hemoglobin and anthropometry every 3 month and serum ferritin at the beginning and end of study (month 1 and 6)

Mean hemoglobin and anthropometry every 3 month and serum ferritin at the beginning and end of study (month 1 and 6)

Figure 3 Proposed trial profiles

# References:

1. Assefa N DM, Zelalem D, Ashenafi W, Dedefo M Profile of Kersa HDSS: The Kersa Health and Demographic Surveillance System. Int J Epidemiol. 2015;27:284.

2. ACS. Addis Ababa, Ethiopia ICF International Calverton. 2012.

3. WHO. Global Database on Child Growth and Malnutrition <http://www.who.int/nutgrowthdb/about/introduction/en/index5.html;> 2016 [cited 2016].

4. Firehiwot Mesfine AW. Anemia among primary school children in eastern Ethiopia. Plose One. 2015;10(4).

5. AmareDesalegn A LG. Nutritional iron deficiency anemia: Magnitude and its predictors among school age children, southwest Ethiopia: A Community Based Cross-Sectional Study. Pubmed Central PMCID. 2014;9(12)(PMC4250059).

6. JenaleeR.doom. Striking while the iron is hot: Understanding the biological and neuro developmental effects of iron deficiency to optimize intervention in early childhood Curr Pediatr Rep 2014;2(4):291-8

7. BaileyRL W B. The epidemiology of global micronutrient deficiencies AnnNutrMetab. 2015 2:22-33.

8. Baumqartner.B. Iron interventions in children from low-income and middle-income populations: benefits and risks. pub Med. 2015; 18(3):289-94.

9. Muyonga J ND NDaDM. Efforts to promote amaranth production and consumption in Uganda to fight malnutrition. In: (Eds) GLRaJRL, editor. Using Food Science and Technology to Improve Nutrition and Promote National Development2008.

10. Catherine W. Macharia DM, Natalie Van den Briel,Agnes M. Omusundi,Alice M. Mwangi,Frans J. Kok,Michael B. Zimmermann,and Inge D. Brouwer. Maize porridge enriched with a micronutrient powder containing low-dose iron as NaFeEDTAbut not amaranth grain flour reduces anemia and iron deficiency in Kenyan preschool children1–3. Journal of Nutrition Community and International Nutrition. 2012;1756.

11. Zebdewos A S, Birhanu G, Whiting SJ, Henry CJ and A Kebebu Formulation of complementary food using amaranth, chickpea and maize improves Iron, Calcium And Zinc content ajfand. 2015 15:4.

12. Ascdrar B. Effect of germination on the chemical composition and nutritive value of amaranth grain. Cereal Chem. 1990;67(6).

13. Price GKOBaML. Amaranth grain & vegetable types. North Fort Mayers,USA ECHO techinical note 1983.

14. Niguse ED. Determinants of anemia among children aged 6-59 months living in Kilte Awulaelo Woreda, Northern Ethiopia. PMCID. 2015.

15. Disha AD RR, Subandoro A and P Menon. Infant And Young Child Feeding (IYCF) Practices in Ethiopia and Zambia Their Association With Child Nutrition:Analysis of Demographic and Health Survey Data. AJFAND. 2012;12:5895-914.

16. L. Alvarez-Jubete HW, E.K. Arendt, E.Gallagher. Polyphenol composition and in vitro antioxidant activity of amaranth, quinoa buck wheat and wheat as affected by sprouting and baking. wwwsciencedirectcom. 2009.

17. Nascimento AC MC, Coelho I, Gueifão S, Santos M, Matos AS, et al Characterization of nutrient profile of quinoa (Cheno podium quinoa), amaranth (Amaranths caudatus), and purple corn (Zea mays L.) consumed in the North of Argentina: Proximate, minerals and trace elements. wwwsciencedirectcom. 2014;148:420.

18. Tibagonzeka J WJ MA, Nakimbugwe D and JH Muyonga. Acceptability and Nutritional Contribution of Grain amaranth Recipes In Uganda. ajfand. 2014;14:3.

19. Rosalind.S.Gibson. Principles of Nutrtional Assessment second ed. New York: Oxford for university press,inc; 2005.

20. Vanessa Dias Capriles ELA REF, José Alfredo Gomes Arêas,Caroline Joy Steel, and Yoon Kil Chang Physical and Sensory Properties of Regular and Reduced-Fat Pound Cakes with Added Amaranth Flour. Cereal Chem. 2008;85(5).

21. Nawiri MP N, Murungi JI. Sun-dried cowpeas and amaranth leaves recipe improves β-carotene and retinol levels in serum and hemoglobin concentration among preschool children. European journal of nutrition. 2013;52(2): 583-9.
